# Unveiling the Conformational Dynamics of the Histone Tails Using Markov State Modeling

**DOI:** 10.1021/acs.jctc.5c00196

**Published:** 2025-04-28

**Authors:** Rutika Patel, Sharon M. Loverde

**Affiliations:** † Ph.D. Program in Biochemistry, The Graduate Center of the City University of New York, New York, New York 10016, United States; ‡ Department of Chemistry, College of Staten Island, The City University of New York, 2800 Victory Boulevard, Staten Island, New York 10314, United States; § Ph.D. Program in Chemistry, The Graduate Center of the City University of New York, New York, New York 10016, United States; ∥ Ph.D. Program in Physics, The Graduate Center of the City University of New York, New York, New York 10016, United States

## Abstract

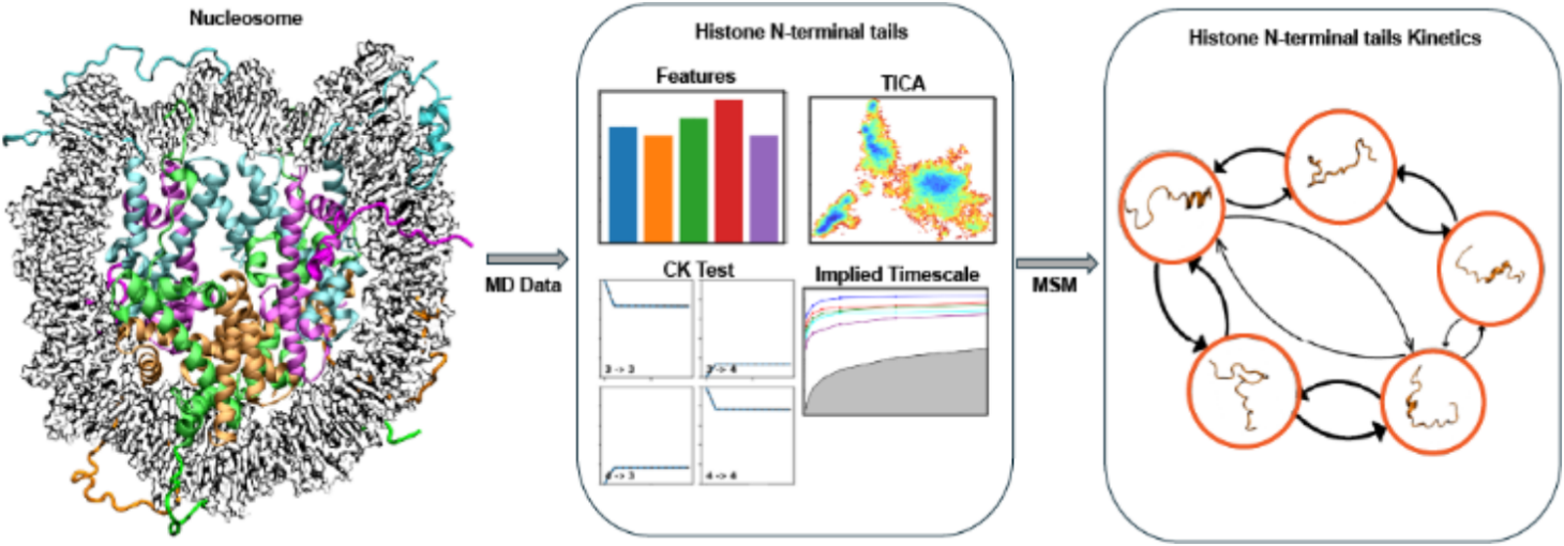

Biomolecules predominantly exert their function by altering conformational dynamics. The nucleosome core particle (NCP) is the fundamental unit of chromatin. DNA with ∼146 base pairs wraps around the histone octamer to form a nucleosome. The histone octamer is composed of two copies of each histone protein (H3, H4, H2A, and H2B) with a globular core and disordered N-terminal tails. Epigenetic modifications of the histone N-terminal tails play a critical role in regulating the chromatin structure and biological processes such as transcription and DNA repair. Here, we report all-atom molecular dynamics (MD) simulations of the nucleosome at microsecond time scales to construct Markov state models (MSMs) to elucidate distinct conformations of the histone tails. We employ time-lagged independent component analysis (tICA) to capture their essential slow dynamics, with k-means clustering used to discretize the conformational space. MSMs unveil distinct states and transition probabilities to characterize the dynamics and kinetics of the tails. Next, we focus on the H2B tail, which is one of the least studied tails. We show that acetylation increases secondary structure formation with increased transition rates. These findings will aid in understanding the functional implications of tail conformations for nucleosome stability and gene regulation.

## 1 Introduction

In eukaryotic cells, DNA is packaged inside the nucleus through a hierarchical structure involving the nucleosome core particle (NCP). Nucleosomes are the fundamental repeat units of chromatin.^1^ Each NCP comprises a histone octamer, around which approximately 147 DNA base pairs are wrapped. Two copies of each of the histones H3, H4, H2A, and H2B assemble to make the histone octamer. Together with histone H1 and linker DNA, they assemble into a higher-order compact and dynamic chromatin structure.^1−6^ Histones have a globular core region and flexible N-terminal tails that protrude from the histone core. The NCP is stabilized by electrostatic interactions between the negatively charged DNA-phosphate backbone and positively charged histone residues, such as lysine (Lys, K) and arginine (Arg, R).^7−9^ Histone tails are major sites of post-translational modifications (PTMs) such as acetylation, methylation, and phosphorylation, which influence the chromatin structure and gene regulation for various biological processes, including DNA repair, replication, and gene expression. PTMs can alter the highly ordered chromatin structure to allow protein modulators to access the DNA.^10,11^ Histone tails are intrinsically disordered. However, the tails are dynamic and can still transiently form secondary structures.^12−15^

The histone N-terminal tails perform several critical biological functions, including internucleosome contacts, nucleosome stability and dynamics, DNA accessibility, nucleosome sliding, and coordinating various epigenetic pathways in time. The tails also speed up the search for nucleosome targets to ease their interactions and are involved in DNA unwrapping.^16^ The histone tails are highly positively charged and interact with the negatively charged DNA-phosphate backbone by the formation of salt bridges. Any changes in the tails via PTMs can perturb these interactions.^16,17^ Each histone tail that protrudes from the histone core has a unique sequence, and the tails are further distinguished by their positioning with respect to the DNA superhelical location (SHL) of NCP. The H3 N-terminal tails (1–43 residues) are located near DNA entry/exit regions and extend between the DNA gyres near SHL ± 7 regions. H3 tails are usually seen collapsed onto DNA rather than extended. Compared to the H3 tails that extend through the entry/exit regions of DNA, H4 N-terminal tails (1–23 residues) are located on the face of NCP, extending from the core near the SHL ± 2 regions. Like the H3 tails, the H4 tails mostly collapse onto the NCP. The H2A N-terminal tails (1–15 residues) are positioned on the face of the nucleosome like H4 tails, but they are farther away from the dyad and located near the SHL ± 4 regions. The H2B N-terminal (1–30 residues) tails protrude between two DNA gyres, and they come into contact with DNA around SHL ± 5 and SHL ± 3 regions. H2B tails are also located close to the H2A N-terminal tails and away from the dyad. The histone tails cover the entire DNA wrap, as tails make contact with every SHL region. Therefore, the tails also play an essential role in DNA breathing and unwrapping.^18^ The truncation of H3 tails has shown a difference in DNA breathing dynamics,^19,20^ a decrease in nucleosome stability,^21,22^ and unwrapping rate.^23,24^ Removal of the H3 and H4 tails increases DNA entry/exit end-to-end distances, suggesting crosstalk between both tails. The tails do not have stable tertiary structures but experience high conformational dynamics at nanosecond time scales.^18,19^

Small-angle X-ray scattering (SAXS) and fluorescence resonance energy transfer (FRET) studies have demonstrated that removing the H3 tail destabilizes the NCP, leading to unwrapping. Also, removing the H4 tails causes DNA to be tightly bound to the histone octamer, indicating that tails distinctly influence unwrapping.^20^ Acetylation increases nucleosome unwrapping, as shown by a single-molecule FRET study.^25^ Several circular dichroism (CD) and nuclear magnetic resonance (NMR) studies have characterized the secondary structure conformations of the histone tails.^26−28^ An NMR study by Kim et al.^29^ has shown that acetylation of H3, H4, H2A, and H2B histone tails causes subtle changes in NCP dynamics. The tail dynamics are observed at picoseconds to nanoseconds time scales. There is an increase in the motions of the acetylated tails and DNA accessibility for regulatory proteins.^29^ In addition, other NMR studies of the NCP have provided insights into tail interaction with proteins.^30−33^ NMR studies have demonstrated that PTMs such as acetylation of the H3 and H4 tails decrease the compaction of nucleosomal arrays to promote the binding of regulatory proteins.^31^ Acetylation of one of the H4 tail residues can also enhance the acetylation rate for the H3 tail, as suggested by NMR studies.^32^ All of the above MD and biophysical methods studies have suggested the histone tail dynamics and effects of acetylation, but the exact effect on the kinetics of these dynamic tails has yet to be understood.

Molecular dynamics (MD) simulations have elucidated some aspects of nucleosome dynamics; however, the kinetics of these processes can be further outlined. Earlier studies of all-atom MD simulations determined DNA conformations,^34^ DNA elastic properties,^35,36^ N-terminal histone tails conformational rearrangement,^37^ nucleosome stability based on H3 and H2B tails DNA interactions,^38−41^ DNA breathing,^42−47^ nucleosome DNA sliding, effects of PTMs of histone H3 and H4 tails that decrease tail-DNA contacts,^48−57^ and DNA unwrapping^44,58^ include the role of histone H2B and H2A tails and DNA loop formation. In addition, MD simulations of the NCP with DNA lesions reveal that the DNA becomes flexible at the lesion site in the complementary strand that faces the solvent, and this can signal for the DNA repair enzymes to recognize the DNA damage.^59^ Another MD simulation study concerning histone H3 hyperoxidation on a single cysteine residue shows that it disrupts the local communication network within the histone core and destabilizes the NCP dyad, suggesting it may promote nucleosome disassembly.^60^ Previous studies conducted by our laboratory included DNA partial unwrapping of the 1KX5 NCP system and wave-like motion promoted by stabilizing effects of H2A and destabilizing effects of H2B tails at microsecond time scales.^61,44^ Another study from our lab demonstrated the motion of the Widom-601 and alpha satellite palindromic NCP DNA sequences at longer microsecond time scales show different pathways for the two sequences, with one showing loop formation and another showing large-scale breathing. The motion and contact of H2A and H2B tails play a critical role in loop formation, and the N-terminal H3 tail plays a key role in the breathing motion of the DNA.^62^ Thus, all-atom MD simulations can aid in characterizing the NCP structure and dynamics.

One of the most well-characterized PTMs is lysine acetylation of the histone N-terminal tail residues. Lysine acetylation neutralizes the positive charge of lysine by replacing it with the acetyl (−CO–CH_3_) group. Histone acetyltransferases (HATs) transfer the acetyl group from acetyl-Coenzyme A (acetyl-CoA) to the ε-amino group of the side chain of lysine residue that neutralizes the positive charge of lysine residues.^29^ Epigenetic modifications are associated with various diseases including cancer, neurological disorders, and inflammatory diseases. One of the histone proteins, H2B, is associated with transcription activation,^63−65^ DNA repair,^66^ and cancer. In addition, one of the tumor repressor proteins, p14ARF, is associated with H2B acetylation at Lys 5, 12, 15, and 20 tail residues. Our previous molecular dynamics (MD) simulation studies showed that the H2B N-terminal tails promote the outward stretching of the SHL-5 region of DNA in the NCP complex in DNA unwrapping.^61^ Our recent study on H2B tail acetylation also revealed that acetylation makes the tail more dynamic and increases helices as well as decreases DNA-H2B tail contacts. It also showed that acetylation rearranges the secondary structure of the H2B tail, making it more helical and changing the conformational space of the tail based on the principal component analysis (PCA).^39^ Histone H2B tail dynamics upon acetylation is a critical factor in biological processes, yet its kinetics remains to be elucidated.

Here, we use Markov State Modeling (MSM) to understand the dynamics and conformational space of histone tails in the NCP system based on molecular dynamics (MD) simulation. Conformational transitions of the histone tails are vital for gene regulation. MSM of molecular kinetics, which approximates the long-term dynamics of molecular systems over a discretized conformational space, has gained extensive applications in recent years.^67,68^ MSMs are used for kinetic analysis by modeling a molecular system as a memoryless transition network, where the probability of transitioning to a future state depends only on the present state, not on the system’s past states. An MSM can describe the entire dynamics of the system. MSM involves an *n x n* square matrix known as a transition probability matrix, where the configuration space spanned by the system is divided into *n* states. The transition probability matrix is characterized by *n* states and by the lag time τ at which the state of the system was recorded. The transition state populations and conditional pairwise transition probabilities can be obtained from this matrix. The resulting models are called transition MSM networks. The transition probabilities yield kinetic information and possible transition state pathways between the states.^68^

Dynamic transitions between metastable conformational states, such as opening and closing of the SARS-Cov-2 spike protein complex, the translocation of RNA polymerases on the DNA template during transcription, etc., are essential to exert their biological functions.^69,70^ There are several MSM and related techniques that have been used to study protein folding^71−78^ and dynamics,^79−81^ protein conformational changes,^82−91^ and protein–ligand binding.^78,92−95^ Histone tails undergo secondary structure conformational changes that include the tail folding and extending into random coil structures. A previous H3 tail MSM study considered only the H3 tail without the DNA of the NCP complex and showed that the H3 tail undergoes conformational changes at nanoseconds time scales.^96^ In addition, MSM and coarse-grained modeling were employed to characterize the complete kinetics of nucleosome assembly using the Widom 601 DNA sequence that identifies various nucleosome conformations based on the kinetic landscapes.^97^ Another coarse-grained modeling study was with a non-Markovian dynamics model, showing the mapping of chromatin folding kinetics and pathways. The findings from this study show that the specific length of the linker DNA is essential to favor chromatin folding in zigzag fibril structures.^98^

Herein, we perform all-atom molecular dynamics (MD) simulations of the NCP at physiological 0.15 M salt concentrations. We run two sets of simulations with one 0.15 M unacetylated (WT) and one 0.15 M acetylated H2B N-terminal tail (ACK) systems. Only the H2B tail residues K5, K12, K15, and K20 are acetylated to study the effects of acetylation on the kinetics of the histone tails. The H2B tail is critical yet not well studied. Our previous H2B tail acetylated study shows changes in the conformational space upon acetylation of H2B tails. We build a MSM of WT H3, H4, H2A, H2B, and ACK H2B N-terminal tails. We perform feature selection using the VAMP-2 scoring method and selected features as a combination of backbone torsions and pairwise distances. Further, we perform dimensionality reduction using time-lagged independent component analysis (tICA) for all histone tails that reveal distinct minima. The kinetic information on the tail is extracted based on the mean first passage time (MFPT) between different transition states of the tails. Comparing the MFPTs between the WT H3, H4, H2A, and H2B tails shows that the H2A tail, being the shortest among the other tails, has the shortest MFPTs. The MFPTs of the acetylated H2B tails are slightly faster than those of the WT H2B tails. Overall, our results establish a clear understanding of histone tail kinetics that adds fundamental insight into nucleosome dynamics.

## 2 Methods

### 2.1 Simulation Methods

The nucleosome core particle (NCP) was simulated at a physiological salt concentration of 0.15 M NaCl. The initial structure configuration of NCP was obtained from the X-ray crystal structure^61^ as reported in the Protein Data Bank (PDB ID: 1KX5). Both subunits of the H2B histone protein N-terminal tails at the K5, K12, K15, and K20 positions of 1KX5 NCP were acetylated by adding an acetyl group using PyMol. The acetylated (ACK) and wild-type (WT) unacetylated tails were parametrized using AMBER force fields. Histone proteins of NCP were parametrized with ff19SB,^99^ and DNA was parametrized using OL15.^100^ The ff19SB is an improved force field compared with the previous ff14SB, and the ff19SB force field includes a CMAP correction. The ff19SB force field employs amino acid-specific CMAP for backbone φ/ψ dihedral angles, which are fitted against quantum-mechanical energy surfaces in an aqueous solution.^99^ Further studies using the force fields developed by Robustelli et al.^101^ for disordered proteins could provide additional insights into tail dynamics. The OPC water model^102^ was used, with its Lennard-Jones interaction (Na^+^/OW) modification, using the Kulkarni et al. method that provides better estimates of the osmotic pressure.^103^ For sodium (Na^+^) and chlorine (Cl^–^) ions, Joung and Cheetham^104^ parameters were used. Mg^2+^ modification was performed using the Li et al. parameter method.^105^ All of the force fields were sourced using the tleap module of AmberTools21 to create the topology and coordinate files for the initial ACK and WT systems. The number of total molecules and water/ions are shown in Table S1.

All systems were initially minimized and equilibrated for 100 ns, followed by production runs for a total of 12 μs. The production runs were carried out using the Anton 2^106^ supercomputer. The minimization was done to reduce unfavorable stress using the conjugate gradient and steepest descent gradient for 40 ps. Following minimization, heating was performed by increasing the temperature of the system to 310 K under *NVT* conditions. Afterward, the systems were equilibrated for 100 ns under *NPT* conditions. The Langevin^107^ dynamics method with the collision frequency with a friction constant of 1 ps^–1^ was used to control the temperature of the system. The pressure of the system was controlled by the Berendsen^108^ barostat. The simulation was continued for production runs under *NPT* conditions with a 2 fs each time step. All simulations used the SHAKE^109^ algorithm to constrain the bonds involving hydrogen. The Lennard-Jones cutoff value for nonbonded interactions was 12 Å, and electrostatic interactions were treated with the particle mesh Ewald (PME)^110^ method with full periodic boundary conditions.

### 2.2 Markov State Model (MSM) Construction

Markov state Models (MSMs) have been a well-known tool in protein folding, ligand binding, and conformational dynamics. To build a Markov model, the features, such as torsion angles, distances, etc., which best represent the slow dynamics of a system are used. Here, we use backbone torsions and pairwise distances of histone N-terminal tails as input features, as we observe that tails undergo secondary structure rearrangement. For both the WT and ACK systems, the backbone torsion angles and pairwise distances as features are selected for MSM construction. The N-terminal residues for the tails are H3 (residues 1-43), H4 (residues 1-23), H2A (residues 1-15), and H2B (residues 4-30).

We use PyEMMA^111,112^ version 2.5.7 for all trajectories to construct MSMs for the N-terminal histone tails of the WT and ACK systems. Time-lagged independent component analysis (tICA) is commonly used for dimensionality reduction^111,113−115^ and is computed for all histone tails with a lag time (τ) of 5 ns. The free energy landscapes of the first two slowest tICA dimensions are plotted for both WT and ACK systems. The free energy landscape is defined as using Δ*G*(*x*,*y*) = −*RT* ln[*P*(*x*,*y*)/*P*_max_], where *P*(*x*,*y*) is the probability density distribution, *R* is the gas constant, T is the temperature (310 K), and *P*_max_ is the maximum probability. tICA Conformational space is segmented into *k =* 200 cluster centers. MSM validation is done using the Chapman-Kolmogorov (CK) test. The CK validation is performed at a lag time of 5 ns for all of the histone tails to show the agreement between the estimated and predicted MSMs. The MSM network with a lag time of 5 ns is constructed using microstates. Microstates are assigned to macrostates using the Robust Perron Cluster Cluster Analysis (PCCA+)^116,117^ algorithm. To identify the transition rates between the conformational states, the mean first passage times (MFPTs) are used, and the inverse of the MFPT provides kinetic rates for each conformational state. In addition, using PyEMMA,^111,112^ we have integrated transition path theory (TPT)^71^ to obtain the net transition pathways and their fluxes. Here, TPT analysis provides a flux network from source state A to sink state B that passes through intermediate states. The dominant pathways of the states and their percentages in total are provided for each system.

## 3 Results

### 3.1 Nucleosome Structure and MSM Overview

**Figure 1:**
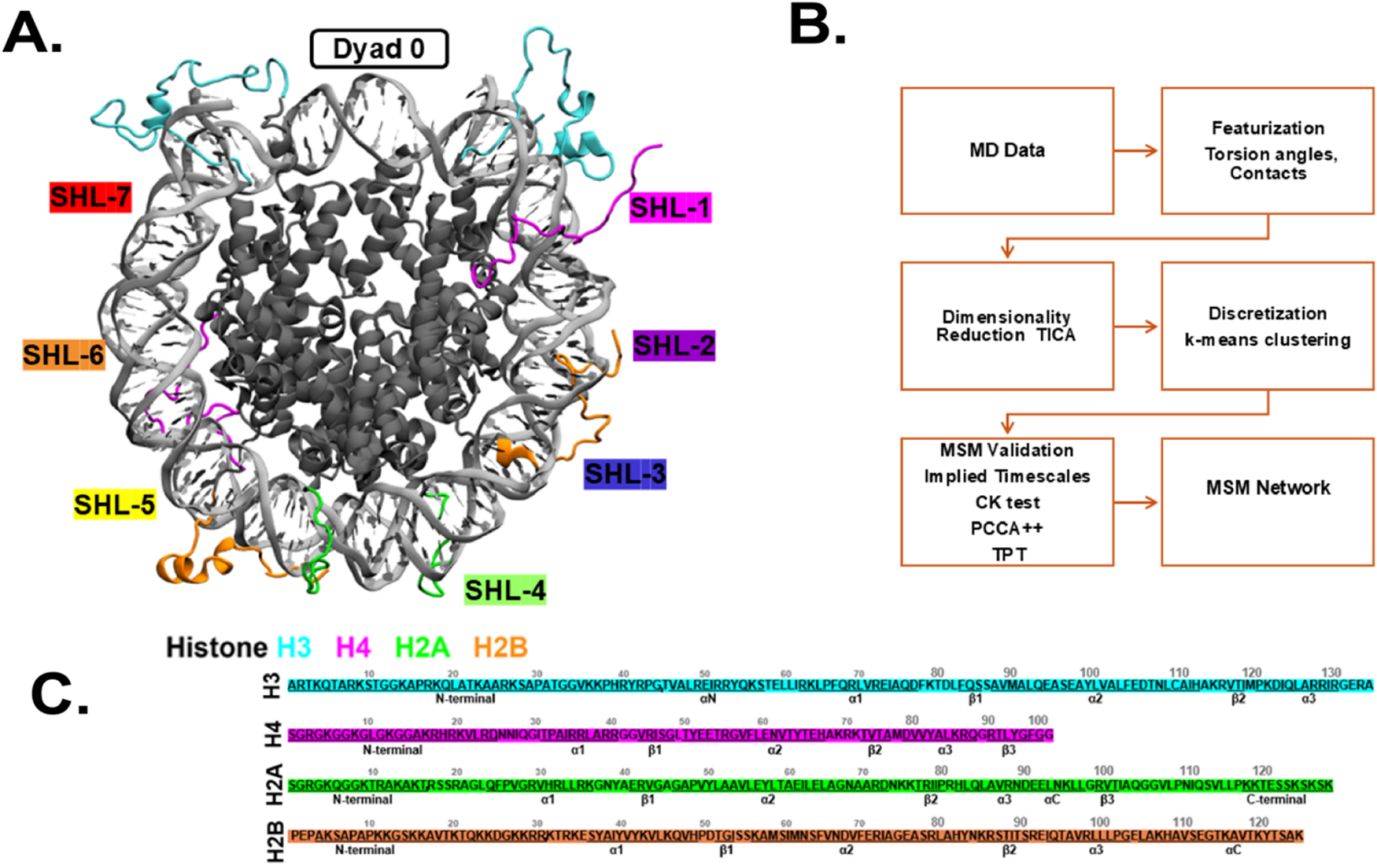
Overview of nucleosome core particle (NCP) structure and MSM workflow. (A) Crystal structure of NCP (PDB ID: 1KX5) consists of 147 DNA base pairs wrapped around two copies of the histone proteins H3 (cyan), H4 (magenta), H2A (green), and H2B (orange). The superhelix locations (SHLs) of DNA are indicated with different colors on the outer side of the NCP. (B) MD simulations are performed, and MD trajectories are processed to select relevant features as input. Dimensionality reduction is performed using the selected features. The dimensionality reduction data are discretized using a k-means clustering algorithm to obtain microstates. Microstates are assigned to macrostates by using the PCCA algorithm. MSM estimation is done based on implied time scales (ITS) and validation through the CK test. The MSM network plot can be generated as a graph where the nodes represent each MSM state. (C) Histone proteins H3, H4, H2A, and H2B sequences with N-terminal residues are shown for all histone proteins. Of all histones, only histone H2B N-terminal tail’s four lysine residues are acetylated.

Here, we perform multiple molecular dynamics (MD) simulations of the nucleosome core particle (NCP) of the 1KX5^61^ system. We simulate wild-type (WT) and H2B tail lysine-acetylated (ACK) systems with four lysines, K5, K12, K15, and K20, acetylated at 0.15 M salt concentration each for 6 μs. We analyze the kinetics of WT H3, H4, H2A, and H2B tails and acetylated (ACK) H2B N-terminal tails by constructing Markov state models (MSMs). The structure of the NCP (PDB ID: 1KX5(61)) system consists of DNA and histone proteins, as shown in Figure 1A. The NCP consists of 147 DNA base pairs wrapped around two copies of the histone proteins H3, H4, H2A, and H2B. The crystal structure of the 1KX5 system was characterized by Davey et al.^61^ at a resolution of 1.9 Å. This system has N-terminal tails resolved in the crystal structure for the histone octamer. The DNA of the NCP has superhelical location (SHL) regions consisting of approximately ten base pairs (Figure 1A). The orientation of the DNA base pairs of the NCP is represented relative to the central base pair, known as SHL zero. The SHL is given where the major groove faces the histone octamer.^118^ The first SHL is SHL 0 at the NCP dyad, and the last is SHL ± 7.

Each histone protein of the NCP has N-terminal tail regions, which are more prone to post-translational modifications. The N-terminal tails do not have a specific tertiary structure, but they undergo changes in the secondary structure conformation. Here, we acetylated four lysine residues (K5, K12, K15, and K20) of both H2B N-terminal tails by adding the acetyl group, neutralizing the positive charge of these lysine residues. Other H3, H4, and H2A N-terminal tails are not acetylated. In our previous study, Patel et al.^39^ have shown that the histone tails undergo conformational changes by transitioning from one conformational state to another throughout MD simulations, and the acetylation of the H2B tail changes the conformational space of the tail. Principal component analysis (PCA) of the H2B N-terminal tails for WT and ACK systems has a free energy landscape with distinct basins belonging to specific N-terminal tail conformations. In another study from our lab, Khatua et al.^62^ also showed that PCA of the H3 N-terminal tails for the WT systems captures distinct conformations of the H3 N-terminal tails. As the N-terminal tails undergo distinct transient conformational changes, the dynamic properties of the histone tails, such as the kinetics of transitions among different conformations, can provide insight into these conformational changes. Therefore, in this study, we use Markov State Models (MSM) to further understand the dynamics of the histone tails in the WT and H2B N-terminal tail acetylated systems. H2B acetylation was chosen since our previous study concerning H2B acetylation demonstrated an increase in acetylated tail dynamics and underwent more considerable secondary structural changes compared to the WT at microsecond time scales. In addition, H2B is a critical gene regulator in cancer and nucleosome dynamics, yet it has not been well characterized. Here, we show the kinetics of WT H3, H4, H2A, and H2B, as well as acetylated H2B tails to characterize the effects of acetylation on the kinetics of the H2B N-terminal tails.

The workflow for the MSM of the MD simulations consists of the steps shown in Figure 1B. First, the features from the raw MD data that represent the dynamics of the system can be extracted. The features include torsion angles, contacts, pairwise distances, coordinates of the protein backbone, etc. Then, the high-dimensional feature space is transformed into a reduced-dimensional space using principal component analysis (PCA) and time-lagged independent component analysis (tICA) to identify key feature components. For MSM analysis, one of the dimensionality reduction techniques is tICA, which constructs linear combinations of the features to identify the reaction coordinates of the slow time scale processes. Further, the MD trajectories are discretized with the tICA space into a state decomposition of the system using the *k-*means unsupervised machine learning algorithm. The *k*-means clustering algorithm segments the reduced tICA space into several cluster centers that group similar conformations. These clusters are known as microstates of the system. Next, MSM estimation is performed to choose a lag time (τ) long enough to ensure Markovian dynamics. Therefore, implied time scales (ITS) as a function of τ can be plotted to select the MSM lag time. The microstates are coarse-grained into a smaller number of macrostates by using the PCCA+ algorithm, and then the MSM validation is performed with the CK test. The CK test can show that the MSM of our system agrees well with the MSM estimated with longer lag times. The transitions between the states are counted and converted to the transition probabilities. The transition probabilities include all transitions between every state including self-transitions. These probabilities can be visualized on the MSM network plot. Other kinetic analyses, such as transition path theory (TPT), can also be performed to observe the flux of probability from the initial state to the final state. In addition, the rates can be measured by calculating the mean first passage times (MFPTs) of the MSM.

### 3.2 Feature Selection and Dimensionality Reduction of Histone N-Terminal Tails

In this work, various analyses are performed based on the explicit solvent simulations of the entire nucleosome core particle (NCP), which is composed of DNA with a histone core and N-terminal tails. To initiate the Markov state modeling, the first step is to extract the features from MD trajectories that best represent the conformational states of the histone N-terminal tails. The set of input features from each instantaneous configuration of the system must be defined to approximate the slow dynamics. As we observed earlier in our previous study^39^ and during our simulations, the histone N-terminal tails undergo secondary structure changes. Therefore, we have selected the internal coordinates of histone tails as backbone torsions and pairwise distance between the C_α_ carbon of amino acid residues of the tails as combined input features to build the MSM. In addition, we have calculated a scalar score obtained by using the variational approach to Markov processes (VAMP) to compare specific features that capture the slow dynamics modes of histone tails. We calculate the VAMP-2 score that maximizes the kinetic variance present in the selected features. We compute the VAMP-2 score for different features for different lag times and find relative rankings of the different features as a function of lag time using PyEMMA^111^ (SI Appendix, Figure S1). The combination of backbone torsions and pairwise distance contains more kinetic variance than other features such as torsions, distance, and inverse distance. This suggests that the combination of backbone torsions and pairwise distance is the best feature evaluated to build the MSM. A previous study about protein folding also constructs MSM by using similar features—combined backbone torsions and pairwise distance as input features.^119^ As the nucleosome has two copies of histone tails, we have analyzed both copies for H3, H4, H2A, H2B, and ACK H2B histone tails to construct the MSM.

**Figure 2:**
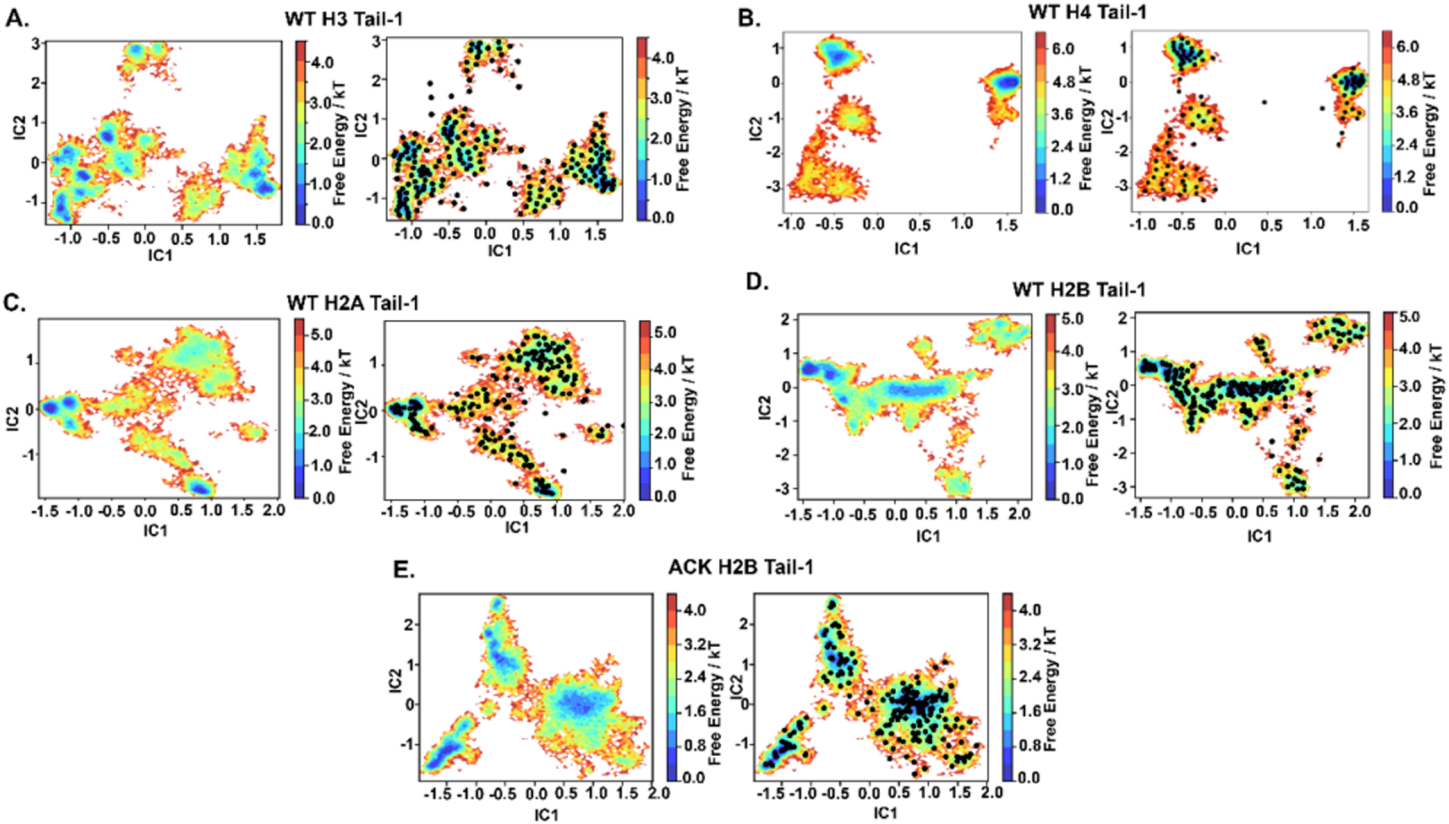
Dimensionality reduction using time-independent component analysis (TICA). The free energy surface and *k-*means clustering visualizations are projected onto the leading two independent components (ICs) for WT (A) H3 tail-1, (B) H4 tail-1, (C) H2A tail-1, (**D**) H2B tail-1, and (E) ACK H2B tail-1. The *k-*means clustering provides microstates for each tail based on their conformational space.

Next, tICA is computed on the features to yield reduced-dimensional space for all histone tails in the WT system, and H2B acetylated tails using PyEMMA.^111^ The free energy projected on the leading two independent components (ICs) exhibits several distinct minima for the histone tails (Figure 2A–E, SI Appendix, Figure S2A–E). As there are two copies of histone tails for each histone protein, we have provided data for all of the histones Tail-1 in the main (Figure 2A–E) and Tail-2 in the SI appendix (SI Appendix, Figure S2A–E). Based on several minima, we assume that our tICA-transformed features describe more than one metastable process in the histone tails. After tICA provides dimensionally reduced data of the MD simulation, these reduced data can facilitate the decomposition of the histone tails system into the discrete Markovian states required for MSM estimation. We use the *k*-means algorithm to segment the tICA space into approximately *k* = 200 cluster centers for almost all histone tails except one of the histone tails, which requires 75 cluster centers. This step groups similar conformations of the WT H3, H4, H2A, H2B, and ACK H2B histone tails into clusters known as microstates.

### 3.3 Markov State Model Estimation and Validation of Histone N-Terminal Tails

**Figure 3:**
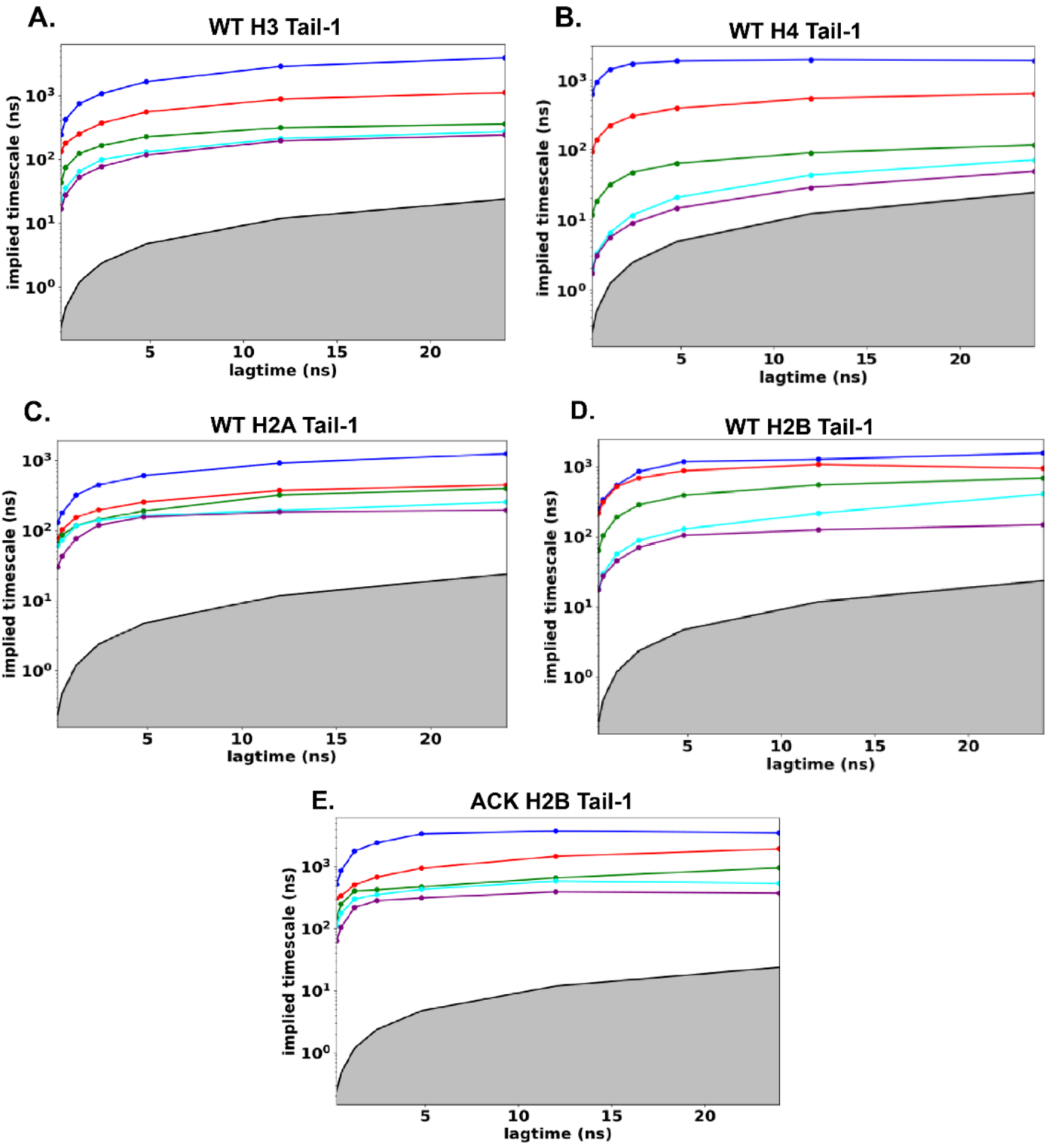
Implied time scales of histone N-terminal tails. The implied time scales (ITS) are associated with the five slowest processes for WT: (A) H3 tail-1, (B) H4 tail-1, (C) H2A tail-1, (D) H2B tail-1, and (E) ACK H2B tail-1. The implied time scale plots show Markov processes with different lag times. The solid line corresponds to the maximum likelihood. The black line with the gray shaded area indicates the time scale horizon below which the MSM cannot resolve processes.

To estimate the MSMs based on our reduced tICA space, the criterion is that the implied time scales (ITs) are approximately constant as a function of lag time τ. Subsequently, the smallest possible lag time τ can be chosen that satisfies this criterion. In the NCP system, the histone N-terminal tails H3, H4, H2A, H2B, and ACK H2B for Tail-1 show five slow processes, and these processes are constant for lag times of >5 ns (Figure 3A–E). We can now estimate an MSM with lag time τ at five ns and perform a validation test. Similarly, for Tail-2 (SI Appendix, Figure S3A–E), all histone N-terminal tails H3, H4, H2A, H2B, and ACK H2B show five slow processes that are constant at different lag times above five ns.

We performed a Chapman-Kolmogorov (CK) test to validate the MSM for the histone tails. Before performing the CK test, an appropriate numbers of metastable states are chosen for the histone tails (SI Appendix Figures S4–S8). For the CK test of the histone tails, the predictions from our MSM (blue dashed line in Figures S4–S8) agree well with the MSM estimated (solid line in Figures S4–S8). The CK tests are performed at the chosen lag time based on the implied time scales that can predict the long-time scale behavior for all histone tails. The CK test for WT H3 (SI Appendix Figure S4) shows four metastable states for Tail-1 and Tail-2, and the prediction from our MSM agrees well with the estimated MSM. Similarly, for histone tail H4, the Tail-1 and Tail-2 CK test shows good agreement for four and five metastable states, respectively (SI Appendix Figure S5). For both H2A tails, four metastable states agree well with our predicted and estimated MSM (SI Appendix Figure S6). For WT H2B Tail-1 and 2, four metastable states are in good agreement as per the CK test, whereas, for acetylated H2B tails, five metastable states for Tail-1 and 2 are in good agreement according to the CK test (SI Appendix Figures S7–S8). All tails show excellent agreement with our predicted and estimated MSM for the CK test, which confirms the choice of metastable states and the lag time.

As mentioned earlier, *k*-means clustering is used to obtain microstates for the histone N-terminal tails; these microstates of histone tails can be clustered to coarse-grain 200 microstates into appropriate four to five macrostates, depending upon the histone tails’ conformational space. The probability of each microstate belonging to metastable macrostates provides the “membership” of each microstate into a few macrostates. For the WT H3 tails and H2A tails, PCCA+ has identified four macrostates. For the H4 Tail-1 and Tail-2, PCCA+ has identified four and five macrostates. For the WT H2B tails, PCCA+ has identified four macrostates, whereas, for the acetylated H2B tails, PCCA+ has identified five macrostates (SI Appendix Figures S9–S10). Next, the MSM is constructed between these macrostates for each of the histone tails.

### 3.4 Markov State Model (MSM) Construction of Histone N-Terminal Tails

Kinetic property analysis can be performed after the estimation and validation of the MSM. So far, to build the MSM of histone tails, the conformational space is divided into a set of discrete microstates, and the microstates are grouped into similar conformations to get macrostates. The MSM network can now be obtained based on the transition *n x n* probabilities to go from one state to another at a specific lag time estimated based on the implied time scales. Each histone tail has about four to five macrostates; therefore, the transition probabilities between these macrostates have been computed to build MSM network plots. We have obtained transition probabilities between the histone tail macrostates for the WT H3 tails, H4 tails, H2A tails, H2B tails, and acetylated H2B tails. These probabilities are plotted on the MSM network plot with the transition probability shown next to the arrow to indicate the transition from one state to another. (SI Appendix Figures S11–S15). After computing the transition probabilities, we also calculated the mean first passage times (MFPTs).

**Figure 4:**
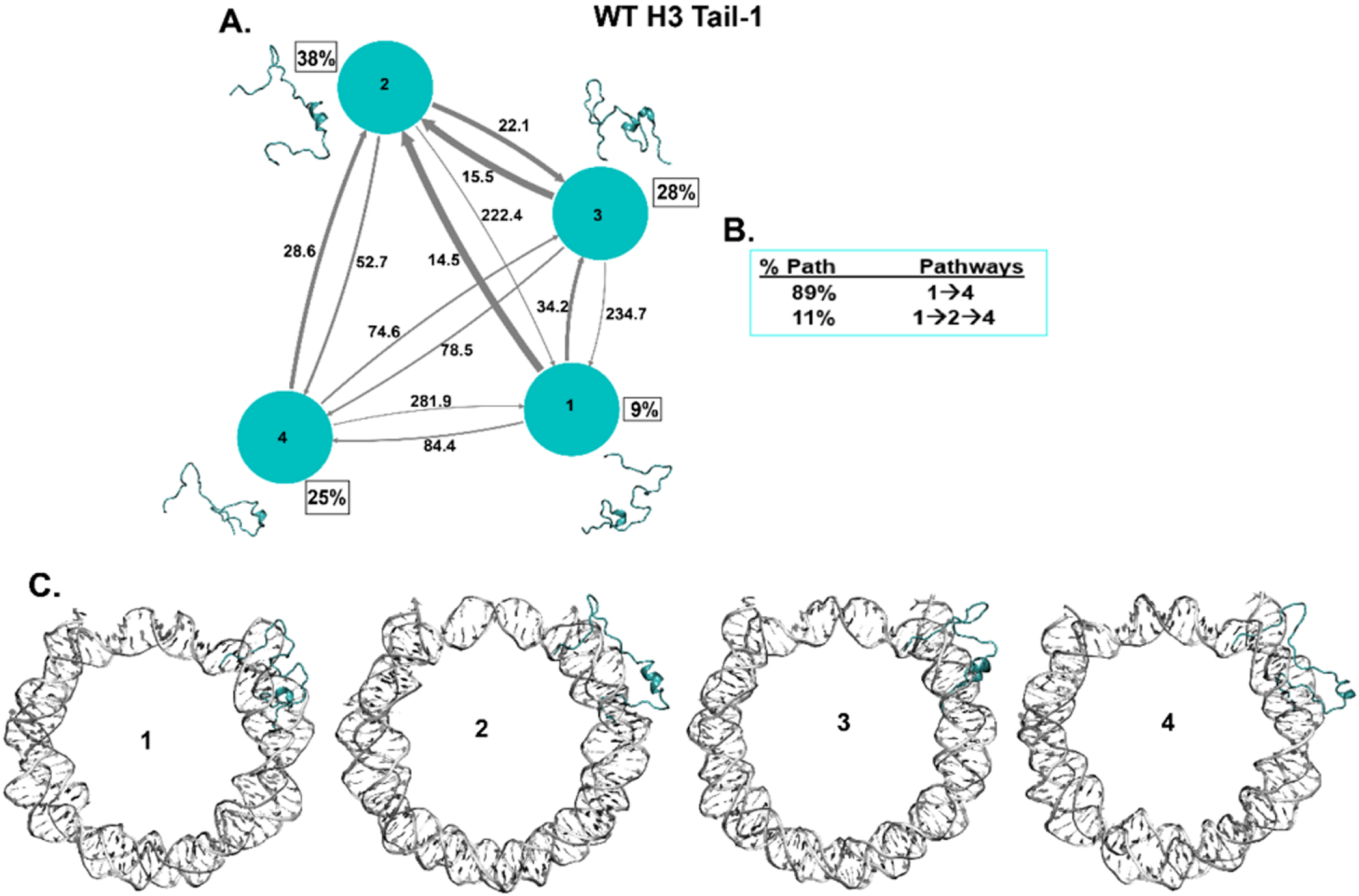
Kinetic network of conformational states of H3 Tail-1. (A) WT H3 Tail-1 (cyan circles) network plots connect the four macrostates of H3 Tail-1. The corresponding conformations of each state are shown next to each state for H3 Tail-1. The population percentages of each conformation are shown next to each state. The macrostates are connected with arrows. The thickness of the arrows is proportional to the transition rate and is labeled with their respective MFPT values in nanoseconds. (**B**) The net flux of the network is obtained from the transition path theory (TPT) analysis for H3 Tail-1. These TPT calculations show major pathways with their path percentages for H3 Tail-1. (C) Conformational states of H3 Tail-1 with NCP DNA are shown. It shows the tail’s position (cyan) at each macrostate with respect to the DNA (silver).

**Figure 5:**
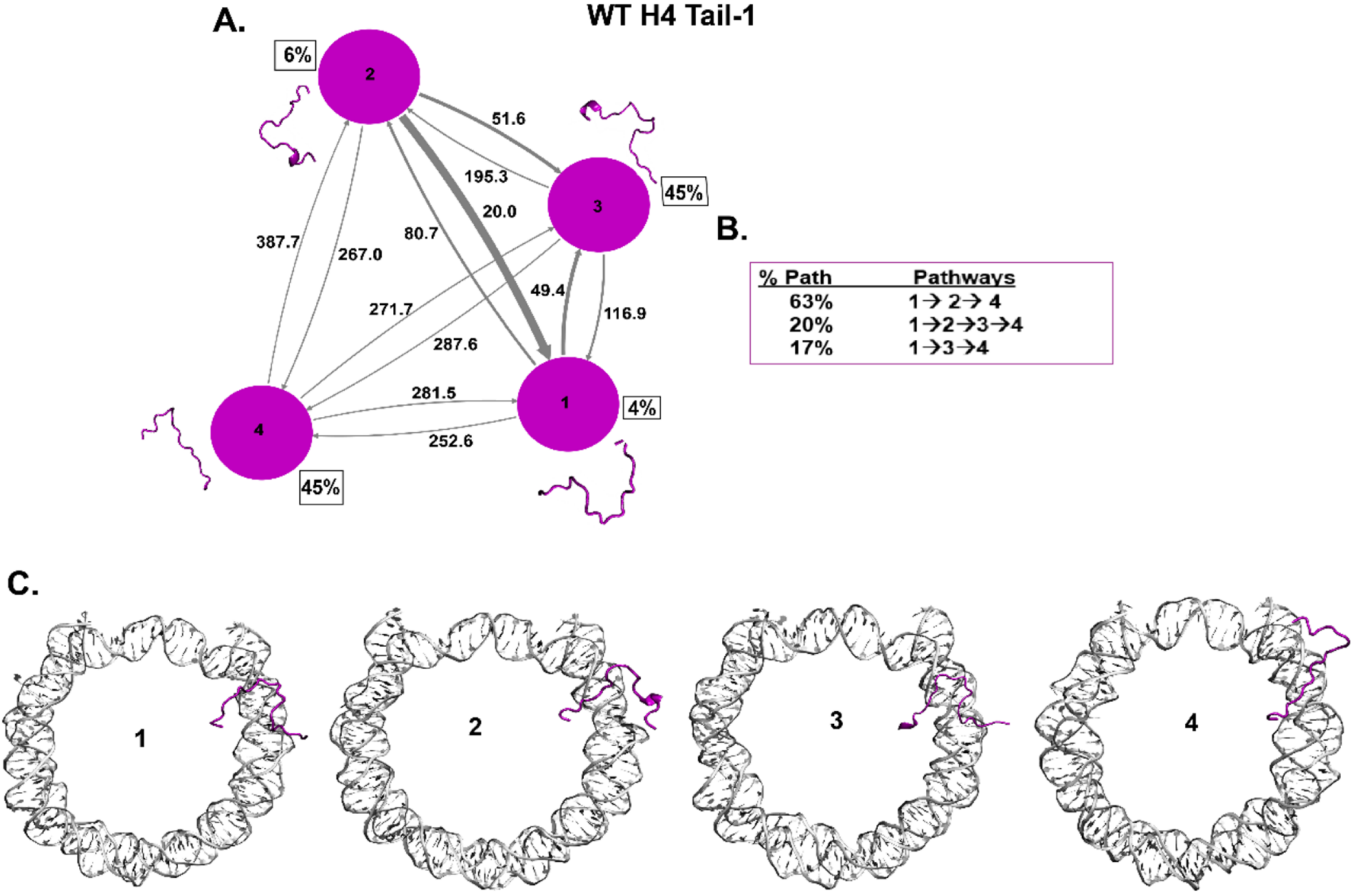
Kinetic network of conformational states of H4 Tail-1. (A) WT H4 Tail-1 (magenta circles) network plots connect the four macrostates of H4 Tail-1. The corresponding conformations of each state are shown next to each state for H4 Tail-1. The population percentages of each conformation are shown next to each state. The macrostates are connected with arrows. The thickness of the arrows is proportional to the transition rate and is labeled with their respective MFPT values in nanoseconds. (B) Net flux of the network is obtained from the Transition Path Theory (TPT) analysis for H4 Tail-1. These TPT calculations show major pathways with their path percentages for H4 Tail-1. (C) Conformational states of H4 Tail-1 with NCP DNA are shown. It shows the tail’s position (magenta) at each macrostate with respect to the DNA (silver).

**Figure 6:**
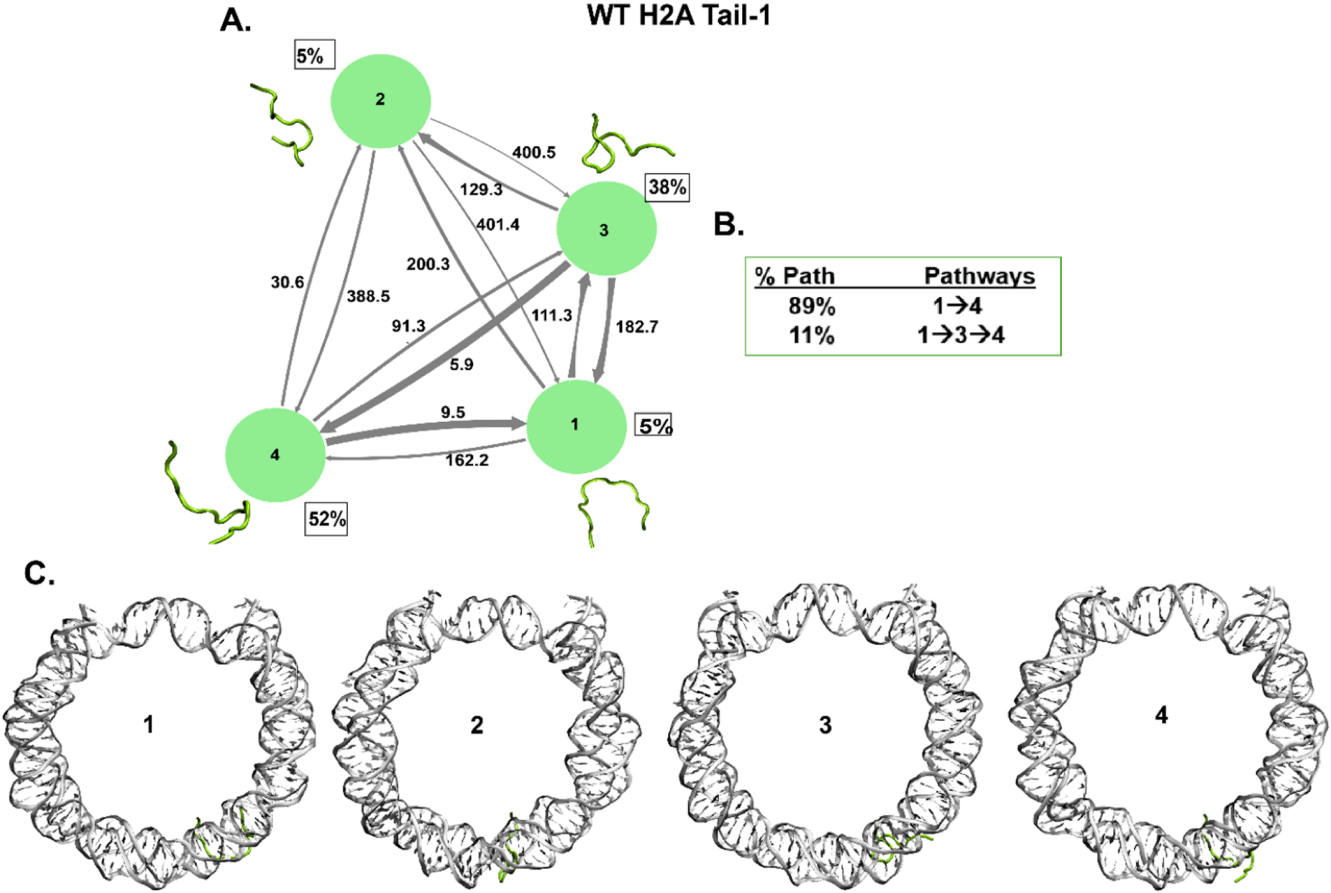
Kinetic network of conformational states of H2A Tail-1. (A) WT H4 Tail-1 (green circles) network plots connect the four macrostates of H2A Tail-1. The corresponding conformations of each state are shown next to each state for H2A Tail-1. The population percentages of each conformation are shown next to each state. The macrostates are connected with arrows. The thickness of the arrows is proportional to the transition rate and is labeled with their respective MFPT values in nanoseconds. (B) Net flux of the network is obtained from transition path theory (TPT) analysis for H2A Tail-1. These TPT calculations show major pathways with their path percentages for H2A Tail-1. (C) Conformational states of H2A Tail-1 with NCP DNA are shown. It shows the tail’s position (green) at each macrostate with respect to the DNA (silver).

**Figure 7:**
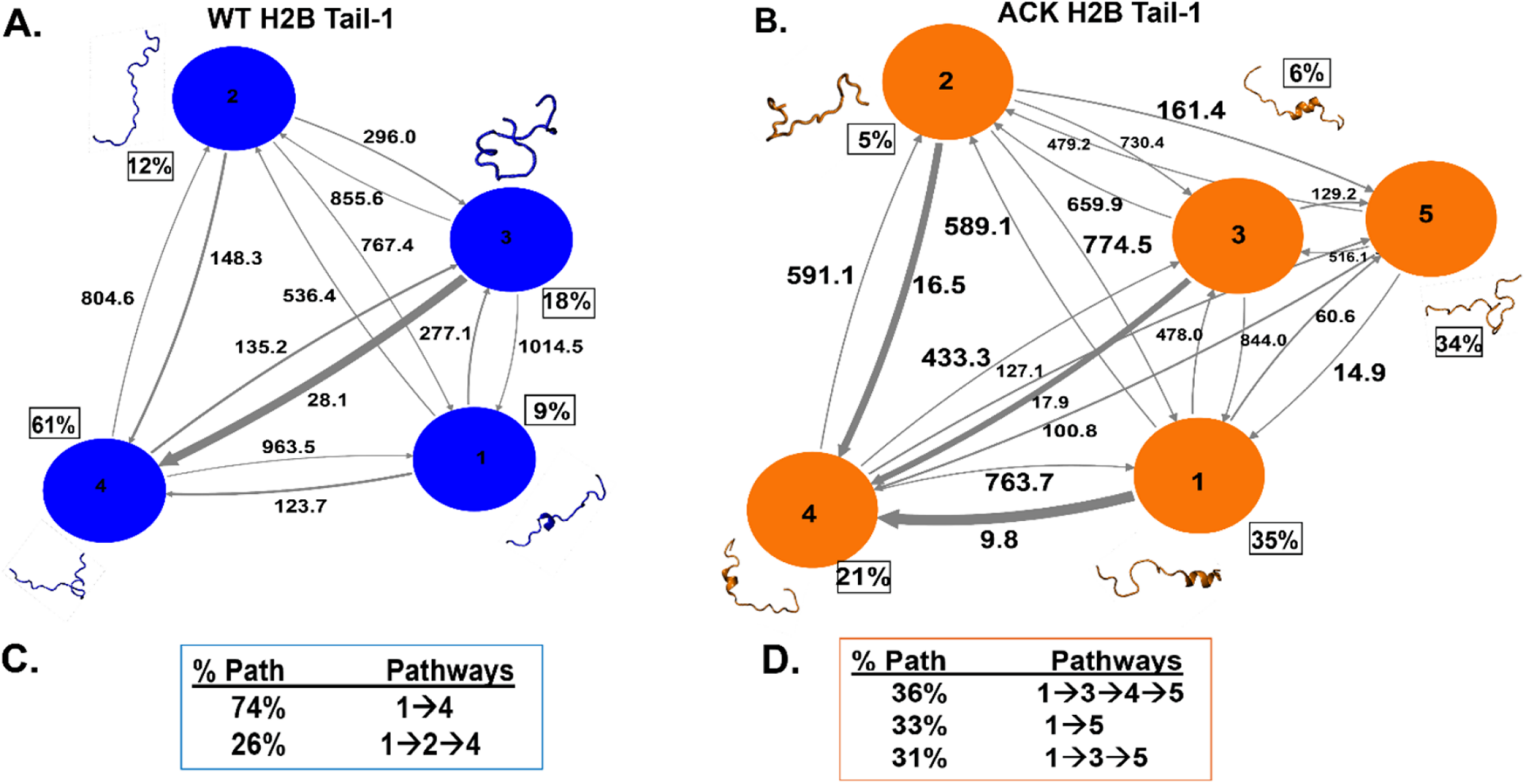
Kinetic network of conformational states of H2B Tail-1. (A, B) WT (blue circles) and ACK (orange circles) H2B Tail-1 network plots connect four and five macrostates, respectively, for H2B Tail-1. The corresponding conformations of each state are shown next to each state for both the WT (blue) and ACK (orange) H2B Tail-1. The population percentages of each conformation are shown next to each state. The macrostates are connected with arrows. The thickness of the arrows is proportional to the transition rate and is labeled with their respective MFPT values in nanoseconds. (C, D) Net flux of the network is obtained from transition path theory (TPT) for both WT and ACK H2B Tail-1. These TPT calculations show major pathways with their path percentages for both the WT and ACK H2B Tail-1 systems.

The MFPTs between the macrostates of the histone Tails-1 (Figures 4, 5, 6, and 7) and Tails-2 (SI Appendix Figures S16–S19) are shown in the nanoseconds range on the MSM network plot. Each histone tail macrostate is labeled with its stationary population percentages. In addition, we have used Transition Path Theory (TPT) to calculate the statistics of the transition pathway between the macrostates of the histone tails with their corresponding percentages. This provides information regarding the TPT fluxes of the significant pathways that histone tails follow.

For the WT H3 Tail-1, the highest population (38%) is state 2, which is a partial helical structure (Figure 4A), whereas Tail-2 shows the highest population, which is state 1 (37%), less helical compared to Tail-1 (SI Appendix Figure S16A). Previous experimental circular dichroism (CD), NMR, and MD simulation studies show that the H3 tail does show some helical structures.^15,26−28,62^ The front part of the tail is slightly disordered for the H3 tails. Also, a mass spectrometry (MS) study indicates that the H3 tails adopt primarily a compact conformation.^121^ It has been seen that H3 tails undergo conformational dynamics at nanoseconds time scales based on NMR and MD simulations.^29,30,96^ The MFPT between all of the states for both H3 tails are at nanosecond time scales, and from both H3 tails, the MFPT is highest at 506 ns and the lowest at around 15 ns. The TPT shows two major pathways of both H3 tails, and the pathway from first to last state 1 → 4 accounts for the majority of percentages compared to other pathways (Figure 4B, SI Appendix Figure S16B). Now, as the NCP structure is composed of both histone proteins and DNA, it is essential to consider the location of each tail conformational state with respect to the nucleosomal DNA. Our previous MD studies have shown that the histone N-terminal tails can collapse onto the DNA or extend away from the DNA. The tails rapidly fluctuate between these two states. Therefore, when we obtained the transition states of the histone tails based on MSM, we also considered the position of all histone H3, H4, H2A, and H2B tails with respect to the DNA, whether collapsed or extended away from the DNA. Both H3 tails mostly stay collapsed or near the nucleosomal DNA SHL ± 7 regions (Figure 4C, SI Appendix Figure S16C). However, we have observed that the tail with partial helical structures stays close to DNA, while random coil structures of the tails collapse on the DNA. The collapse of the H3 tails conformations is consistent with previous observations of direct interactions of collapsed H3 tails with the nucleosomal DNA based on NMR single-molecule FRET and MD studies.^30,33,62,122,123^ A previous MD study from our lab also demonstrated that H3 tails condense in the minor groove on the SHL ± 7 region and observed helical conformations of the H3 tails.^62^ The H3 tail pathways based on the TPT show that tails undergo partial helical to slightly less helical, but the front of the tail mostly stays collapsed onto nucleosomal DNA.

Further, histone WT H4 Tail-1 shows the highest population (45%) for two states with random coil and turn structures (Figure 5A), whereas Tail-2 shows the highest population (39%) for state 4, which is a random coil structure (SI Appendix Figure S17A). The TPT shows three significant pathways for both H4 tails (Figure 5B, SI Appendix Figure S17B). H4 Tail-1 shows 1 → 2 → 4 as a major pathway, while Tail-2 shows 1 → 4 as a major pathway between macrostates. The Tail-1 interconverts from random coil to turn and has a slight helical structure. In addition, the MFPT between different states is shown in nanoseconds time scales, with 15 ns being the lowest and 387 ns being the highest MFPT among both H4 tails. Also, H4 Tail-2 shows predominantly random coil conformations and a slightly faster MFPT than that of Tail-1. H4 Tail-1 has tail conformations that mostly collapse on the nucleosomal DNA near the SHL ± 2 region; the H4 Tail-2 has random coil conformations that stay collapsed between the two DNA gyres (Figure 5C, SI Appendix Figure S17C). Previous NMR and HDX-MS studies show that H4 tails conformational dynamics occur at nanosecond time scales.^29,31,124^ In addition, the H4 tails conformational dynamics of its basic patch interact with adjacent DNA and H2A/H2B acidic patch in a nucleosome array.^125−127^

The histone H2A N-terminal tail is the shortest (1–15 residues) among the histone tails of the NCP. Both H2A tails show random coil structures. WT H2A Tail-1 shows the highest population (52%) for state 4, a random coil. The other states are also random coils (Figure 6A). For H2A Tail-2, the highest population (50%) is state 4, which is also a random coil, and other states of Tail-2 are random coils as well (SI Appendix Figure S18A). H2A Tail-1 and Tail-2 show major pathways as 1 → 4 out of two pathways, and all in-between states are random coils (Figure 6B, SI Appendix Figure S18B). The MFPT between different states shows nanosecond time scales, with around five ns being the lowest and 415 ns being the highest among H2A tails. Also, NMR and MD simulation studies showed that H2A N-terminal tails are unstructured and dynamically faster at nanosecond time scales.^15,31^ Further, both H2A tails collapse on the DNA between the two DNA gyres (Figure 6C, SI Appendix Figure S18C). The H2A tails collapse around SHL ± 4 regions and interact with nucleosomal DNA, consistent with the previous studies.^24,128,129^

### 3.5 Effects of H2B N-Terminal Acetylation for MSM Analysis

The MFPTs between the WT and ACK H2B Tail-1 macrostates are shown in the nanosecond range on the MSM network plot (Figure 7A–B). The highest population (61%) for the WT H2B Tail-1 is state 4, a random coil structure, whereas the highest population (35%) for the ACK H2B Tail-1 is state 1, a helical structure. Similarly, for H2B Tail-2, the highest population for WT H2B Tail-2 is state 4 (56%), and for ACK H2B Tail-2 is state 5 (40%) (Figure 7A–B, SI Appendix Figure S19A–B). Both states show random coils. However, acetylated H2B Tail-2 involves more helices in the H2B tail than in the WT H2B Tail-2. Thus, ACK H2B Tail-1 and Tail-2 involve more helix propensity than WT. We also observed this phenomenon in our previous study, where there was an increase in helicity in the H2B tail upon acetylation.^39^ In addition, the experimental study by Wang et al.^27^ has described that lysine acetylation increases the helical content of the histone tails by performing circular dichroism (CD) analysis.^27,57,130^

**Figure 8:**
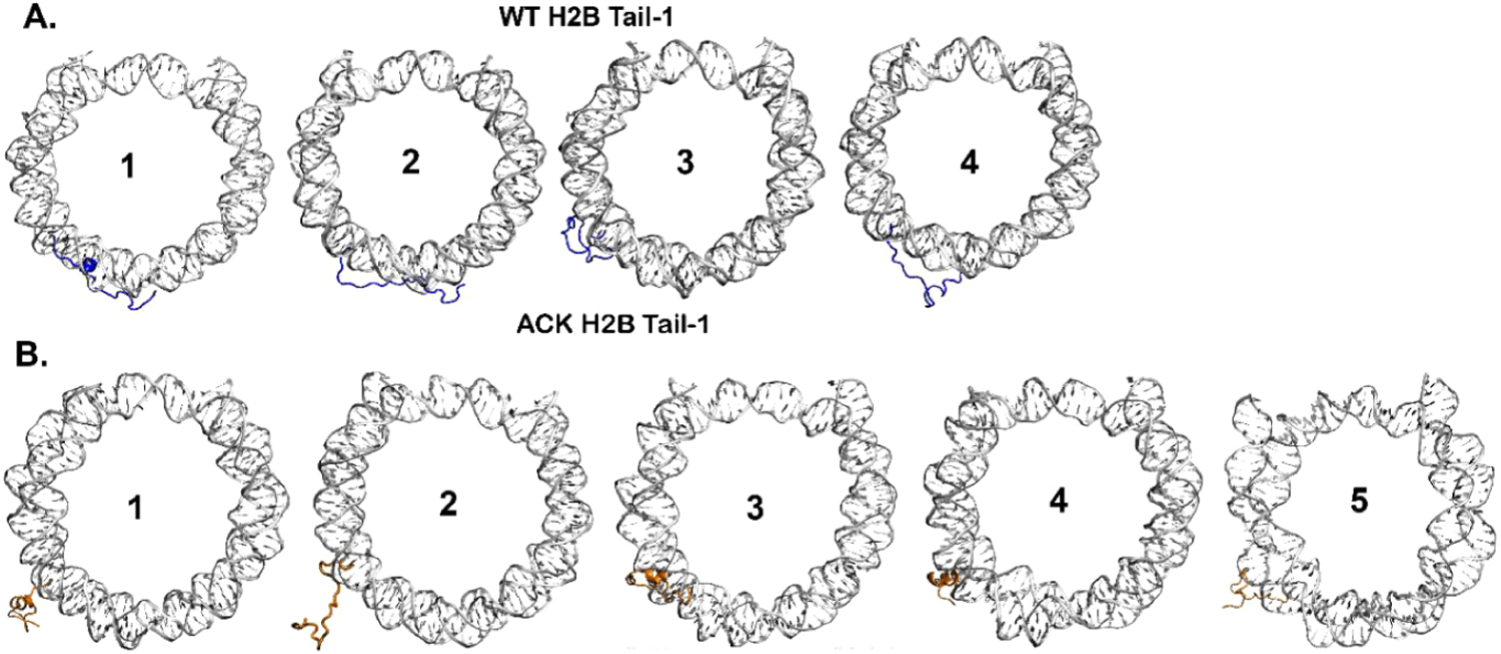
**C**onformational states of H2B Tail-1 with the DNA of NCP. (A, B) WT (blue) and ACK (orange) H2B Tail-1 conformational states are shown in Figure 4; the same conformational states of the tail with NCP DNA are shown here. It shows the position of each tail conformation at each macrostate shown earlier with regard to the DNA, whether the tail collapsed to the DNA or elongated outwards from the DNA.

Further, we compute the TPT flux between macrostates (Figure 7C–D, SI Appendix Figure S19C–D). We calculate the major pathways between the states of the H2B tails for both the WT and the ACK systems with their corresponding percentages. This provides information regarding the major pathways that histone tails follow to exert their functions. Earlier, we showed that histone N-terminal tails can collapse onto the DNA or extend away from the DNA during MD simulations. The tails rapidly fluctuate between these two collapsed and extended conditions. In acetylation, the tails mainly extend away from DNA.^39^ Therefore, when we obtained the states of the histone H2B tails for WT and ACK based on MSM, we also considered the position of each conformation, whether they are collapsed or extended away from the DNA for H2B Tail-1 and Tail-2 (Figure 8A–B, and SI Appendix Figure S20A–B) for both the WT and ACK systems. As the acetylated H2B tails have four acetylated lysine residues, it shows that three out of five states of the H2B Tail-1 extend away from the DNA. Most of the interactions of each histone tail bound to the DNA are due to strong electrostatic interactions via salt bridge formation between the positively charged amino acids of the tails and the negatively charged phosphate backbone of the DNA. When the lysine residues of the H2B tails are acetylated, the H2B and DNA interactions decrease due to the charge neutralization of the lysine residues. As the interactions break between the tail and the DNA, the H2B tail extends out and stays away from the DNA. As shown in Figures 8B and S20B, most acetylated H2B tail conformations extend out and stay away from the DNA compared to the WT. For example, the acetylated H2B Tail-2 state 5, which shows a majority population of 40% (SI Appendix Figure S19B), stays away from the DNA (SI Appendix Figure S20B). The H2B Tail WT state 1, which shows the majority population of 56%, stays close to the DNA and between the two DNA gyres around SHL ± 5 and SHL ± 3 regions. Similarly, for acetylated H2B Tail-1, the first state with the highest population is helical and extended away from the DNA (Figures 7B and 8B).

The kinetic network shows multiple pathways via TPT of WT and ACK H2B Tail-1 and Tail-2 (Figure 7C–D, SI Appendix Figure S19C–D). We have identified the pathway for the WT system for H2B Tail-1 that accounts for 74% (1 → 4) of the total pathways, and the majority of these transition states from first to last states of the H2B tails that either collapse onto the DNA or are closer to DNA around SHL ± 5 and SHL ± 3. In this major pathway, the H2B Tail-1 for WT that was initially in between two DNA gyres has slightly moved out of two gyres yet stayed in proximity to the DNA. For the acetylated H2B Tail-1, there are three pathways, and one of the pathways 1 → 5 shows a change in the secondary structure conformation, and the tail stays away from the DNA. The intermediate transition states from the first to the last state show tail fluctuation between the collapse of the DNA and the extension away from the DNA. As not all positively charged lysine residues of the H2B tails are acetylated, other positively charged residues still attempt to interact with DNA. For WT H2B Tail-2, the major pathway that accounts for 84% (1 → 4) of the total pathways, shows the tail location that is present between DNA gyres shifts in closer proximity to the DNA. The ACK H2B Tail-2 is the major pathway 1 → 3 → 5, the tail conformation shifts from helices to random coil and stays away from the DNA. As the DNA has SHL regions from SHL 0 to SHL ± 7, the H2B tails mainly interact with the SHL ± 5 and SHL ± 3 regions. Therefore, when the tail conformations collapse onto the DNA or stay near it, they primarily interact with the SHL ± 5 and SHL ± 3 regions.

## 4 Discussion

Herein, we aim to elucidate the kinetic properties of the conformational dynamics of histone tails H3, H4, H2A, H2B, and acetylated (ACK) H2B. This can provide insights into how the kinetic properties of the tails can be correlated to their biological functions. We perform a total of 12 μs long all-atom MD simulations of two NCP systems (PDB: 1KX5) at 0.15 M salt concentration of unacetylated (WT) and H2B tail lysine-acetylated (ACK) systems. We parametrized the histone proteins of the NCP using ff19SB,^99^ and DNA was parametrized using OL15^100^ force fields. The ff19SB is an improved force field from the previous ff14SB, and it includes a CMAP correction fitted against quantum mechanics. It uses a 2D grid-based energy corrections that improve the accuracy of conformational landscapes.^99^ In the future, we can explore a99SB-disp force field for disordered proteins by Robustelli et *al*.^101^ Previous NCP studies have used ff14SB, ff99SB, and modified force fields for the N-terminal tails.^15,131−133^ The acetylated lysine residues for both H2B tails are Lys 5, 12, 15, and 20 at a 0.15 M salt concentration. The degree of acetylation of the H2B tail is approximately 40%. These particular lysine residues are associated with the tumor repressor protein p14ARF^134^ and ATF2^135^ coactivator. Both p14ARF and ATF2 proteins maintain transcription through H2B tail interactions and acetylation during gene regulation. The interaction of p14ARF with H2B tails involves deacetylation via HDAC1, leading to transcription repression, and in the absence of p14ARF, HAT acetylates these four lysine residues, leading to transcription activation. The p14ARF controls apoptosis or cell death in response to oncogenic stress and regulates gene transcription; however, p14ARF is often mutated in many human cancers. In addition, ATF2 (activating transcription factor 2) has been associated with H2B and H4 acetylation. It is a coactivator for p300 HAT, but ATF2 also has HAT activity that acetylated the H2B tail lysine at positions 5, 12, and 15, which results in transcription activation. The H2B N-terminal tail is a critical regulator in gene expression and is ubiquitous in diseases, yet the dynamics and kinetics of the H2B tails remain to be understood.

Histone N-terminal tails are disordered yet undergo transient secondary structure conformation changes. Our previous study, Patel et al.^39^ showed that the H2B histone tails undergo conformational changes by transitioning from one state to another throughout MD simulations. Using principal component analysis (PCA) of the H2B N-terminal tails for WT and ACK systems, we show distinct free energy basins belonging to specific H2B tail conformations. The DNA-histone tail interaction decreases and solvent accessible surface area (SASA) for histone H2B tails increases upon acetylation. The dynamic properties of histone tails, such as the kinetics of transitions among different conformations, need to be further elucidated. One computational study by Zheng et al.^120^ focuses on the H3 tail free energy landscape and underlying kinetics. Still, this study considers only H3 tails, and other tails with the entire NCP remain to be elucidated. They use input features such as dihedral angles and show that the H3 tail rapidly transitions between different states with their MFPTs.^120^ It is essential to consider the tails in complex with the DNA of the NCP to understand the tail dynamics. Here, we use Markov state models (MSM) to further understand the dynamics of histone tails H3, H4, H2A, H2B, and ACK H2B in the full NCP complex. A summary of the major findings for each histone tail is in Table 1.

**Table 1:** Summary of Histone Tail Conformational Dynamics and Kinetics Analysis

**histone tails**	**highest population**		**structural conformation**	**MFPT** (**ns**)		**total no. of states**	**major transition path**
	**state**	**%**		**low**	**high**		
WT H3	2	38%	partial helical	15	506	4	1→4
WT H4	3	45%	minor helix/turn	15	387	4	1→2→4
WT H2A	4	52%	random coil	6	415	4	1→4
WT H2B	4	61%	random coil	18	1014	4	1→4
ACK H2B	1	35%	helical	4	763	5	1→3→4→5

We observe that histone H3 tails and H2B tails show a slightly more helical structure of the tails compared to the H2A and H4 tails based on our MSM analysis. H3 and H2B tails have approximately 30% hydrophobic residues, whereas the H2A and H4 tails have only about 15%. Helical or globular proteins contain about 50% hydrophobic residues, such as phenylalanine, leucine, and isoleucine, with bulky hydrophobic groups.^136^ The H3 tails are mostly collapsed onto the DNA near the SHL ± 7 regions. We extract microstate conformations for each macrostate. The superimposed microstate structures provide insight into the conformational variability within that particular state (SI Appendix Figures S21–S25). We show the H3 tail conformations and their conformational variability for all H3 Tail-1 and Tail-2 macrostates in SI Appendix Figure S21. Collapse leads to interactions with DNA and NCP core regions. These contacts are primarily driven by the positively charged lysine and arginine residues of the H3 tails.^30,33,62,122,123^ Our previous MD study has shown that when H3 tails collapse onto DNA, it leads to the outward movement of the SHL ± 7 region. This results in unwrapping of the DNA entry/exit region, initiating the breathing motion. Upon collapse, the helical content of the tail increases.^62^ Conformational changes of the H3 tail could facilitate binding to the PHF1 Tudor domain, promoting partial unwrapping of the DNA. This indicates that the H3 tail can regulate DNA binding dynamics.^137^

H4 tail conformations are slightly less helical than H3 tails, but like H3 tails, H4 tails also mostly collapse onto the DNA around the SHL ± 2 region. The H4 tail microstate conformations and their conformational variability for all H4 Tail-1 and Tail-2 macrostates are given in SI Appendix Figure S22. One of the H4 tails shows a slightly shorter MFPT than that of the other H4 tail. It has been suggested that there is crosstalk between H3 and H4 tails, which might arise from changes in the conformational ensembles of one tail’s DNA collapsed/bound state when the other tail is perturbed.^18,19^ H4 tails interchange their conformations between random coils to slightly helical. As mentioned, conformational ensembles are part of crosstalk; the kinetics of tail conformations can provide valuable insights into the mechanisms of their interaction and dynamics. H4 tail residues 16–23 include mostly positively charged lysine and arginine, known as the H4 basic patch, located close to the NCP core. The conformational dynamics of the H4 tails basic patch forms intra- and/or internucleosome interactions with adjacent DNA or the H2A/H2B acidic patch in a nucleosome array.^125−127,138^

H2A tails are the shortest compared with the other histone tails. This could lead to an increase in their fluctuations with a lower MFPT. Among all of the WT histone tails, H3, H4, H2A, and H2B, the WT H2A tails show the lowest MFPT. H2A tails exhibit predominantly random coil conformations of the tail. The tails are also collapsed around the SHL ± 4 regions. Microstates of the H2A tails conformations and their conformational variability for all macrostates of both H2A Tail-1 and Tail-2 are shown in SI Appendix Figure S23. Just like how the H3 tail’s collapse onto the DNA contributes to unwrapping, H2A tails are also linked to promoting DNA unwrapping.^44,123^ Certain histone tails may influence the dynamics of other tails. In particular, the H2A N-terminal tails are positioned close to the H2B tails and might influence each other. Notably, the correlation of the H3 and H2A tails can modulate DNA breathing dynamics.^18^

Based on the network plot of WT and ACK H2B tails, acetylated ACK H2B tails mostly have α-helix conformations compared to the other WT tails. Computational and experimental studies have observed that acetylation increases the helicity in the histone tails.^27,39,139^ WT H2B tails are predominantly random coils compared to ACK tails. This might be due to the H2B tails being highly positively charged with two Arg and ten Lys residues, which generally creates a repulsive electrostatic interaction that makes it harder to form distinct compact helical structures. Upon acetylation, the repulsion decreases in the tail, as there is charge neutralization of the acetylated lysine residues. Adding the bulky acetyl group would create less repulsion and more hydrophobicity, shifting the tail into compact helical structures. Thus, we observe more helicity of the tail in ACK H2B Tail-1 and Tail-2 compared to WT.

Epigenetic modifications, such as acetylation in chromatin, regulate vital cellular processes such as transcription, DNA damage repair, and gene regulation. Epigenetic modifications are closely linked to diseases such as cancer, neurological disorders, and inflammatory diseases. Acetylation is closely linked to transcription, as it makes chromatin transition from tightly packed to loosely packed states, making the NCP nucleosomal DNA more accessible to RNA polymerase II, transcription factors, and other proteins required for gene regulation. It can also help prevent specific proteins from interacting and causing DNA damage and is involved in DNA repair. Acetylation of H2B tails leads to faster rates and more dynamic tails; this was also observed in a previous NMR study. Our MFPT rates are in nanosecond time scales, which is rapid for acetylation. This agrees with the NMR study that observes the tail conformational changes at the rapid time scales of picoseconds to nanoseconds upon acetylation.^29^ Although it is often challenging to study tail dynamics using biophysical techniques, one of the solid-state NMR studies has shown that acetylation during PTMs of histone tails leads to an increase in tail dynamics and, subsequently, stronger interactions with other proteins.^29,33^ Furthermore, the rapid transitions in the acetylated tail may regulate the access of key proteins to nucleosomal DNA. Previous NMR studies have shown that acetylation increases enzyme activity as DNA becomes more accessible for enzymes to conduct biological functions. For example, the acetylation of histone tails, specifically H2B and H2A, causes an increase in ligation with LIG3 (DNA Ligase 3) and fluctuation of H2B tails.^29^

Further, the H2B tails in the ACK system have shown compact helical structures upon acetylation. This could be a docking site for other proteins that recognize the acetylated sites. Bromodomains (BRDs) are proteins that recognize multiple acetylated lysine residues at the site of the histone tails. As histone tails become more hydrophobic, they can fit perfectly in the hydrophobic binding pocket of BRD. The BRD is associated with epigenetic reader modules and has been implicated in drug treatment for cancer diseases.^52,140−142^ Most of the conformational populations for the H2B acetylated tails are helical conformations compared to those of WT based on our MSM. We use transition path theory (TPT) to characterize the transition state pathways for the WT and ACK H2B tails. The histone tails fluctuate between collapsing onto the DNA near the SHL ± 3 and SHL ± 5 regions and extending away from the nucleosomal DNA. The tail conformations of the macrostate in the ACK system mostly show the tail extending away from the DNA. The tail conformations of the ACK H2B tails stay in proximity to DNA in helical structures. These conformations may facilitate docking to other proteins to access the nucleosomal DNA of the NCP. The microstates of the H2B tail conformations are shown for all macrostates of both H2B Tail-1 and Tail-2 in SI Appendix Figures S24 and S25. Other histone tails, H3, H4, and H2A, show tail conformations that are mostly collapsed onto the DNA or are present between the two DNA gyres.

The histone tails have been implicated in assembling higher-order chromatin structures, including internucleosome interactions, nucleosome array compaction, and array oligomerization. H4 tail interactions with an acidic patch of the adjacent nucleosomes in a tetranucleosome show that the H4 tail interacts with the acidic patch and at the interface between tetranucleosomes forms stable nucleosome array structures. This also provides the importance of tail collapse to the DNA making DNA-tail contacts. In addition, previous MD studies show that all of the tails form internucleosome contacts primarily through DNA-tail interactions.^18,138,143^ Also, when the tail is modified through PTM, such as acetylation, the conformational ensemble of the tail becomes more compact, promoting intranucleosomal interactions rather than internucleosomal interactions.^52,144^ As we observed, H2B acetylation shows faster MFPT; this can facilitate a quick transition from inter- to intranucleosome interactions in regulating higher-order chromatin structure. The faster transition between states can also facilitate quick searching for new binding proteins as the tail decreases its interactions with nucleosomal DNA.^18^

As we have observed, histone tails alter their conformations, and upon acetylation, tails alter their conformations compared to those of WT. These conformations may also contribute to crosstalk. The conformational ensemble of one tail may alter the adjacent tail. As the H2B and H2A tails are closer, the changes to the H2A tail may alter the H2B conformations. Both tails interact with DNA SHL regions that are closer, so the changes to one tail’s DNA interactions may alter the neighboring tail. A recent study found that acetylation of the H4 tail can affect the conformational dynamics of the H3 tail^18,32^The tail conformations are likely a critical contributor to chromatin PTM crosstalk. Therefore, studying the kinetics of the tail conformations can also provide insight into crosstalk.

## 5 Conclusions

In conclusion, we perform long-time all-atom molecular dynamics simulations of the NCP at a 0.15 M NaCl concentration for WT (unacetylated) and ACK (acetylated H2B tail) systems. The four lysine residues, K4, K12, K15, and K20, of both H2B tails are acetylated. As histone tails are dynamic, the kinetics of these tails needed to be elucidated. Here, we analyze the kinetics of the histone H3, H4, H2A, and H2B tails and ACK H2B tails to assess the structural dynamics of the histone tails. Our results highlight that acetylated H2B tails show faster rates based on their mean first passage times (MFPT) compared to WT systems. Here, the MSM of histone tails is constructed under physiological conditions. If physiological conditions, such as temperature or salt concentration, change, the histone tail rates may be affected. At higher temperature conditions than physiological, the rates between the states of histone tails may increase.^145^ A study by Wei et al.^146^ demonstrated that salt concentration and DNA-histone interaction influence DNA unwrapping and rewrapping kinetics. The study shows that higher salt concentrations increase the DNA unwrapping and rewrapping rate. As the salt concentration increases, the dynamics of DNA change, leading to higher rates of nucleosomal DNA unwrapping. We expect a similar pattern for the histone tails at higher salt concentrations as DNA unwrapping is linked to histone tails through protein–DNA interactions. Our current observations based on the MSM outlined here align well with a previous NMR study^29^ that reports increased dynamics of the tails upon acetylation. H2B tail acetylation leads to the tail’s structural changes, making it more helical, which can serve as docking sites for other proteins. Acetylated H2B tails also show more fluctuations in tail motion compared to those of WT; rapid fluctuations of the ACK tails support regulatory protein activity for biological functions and nucleosome plasticity.

## Data Availability

Analysis codes are available on the GitHub Repository. https://github.com/CUNY-CSI-Loverde-Laboratory/Histone_N_terminal_Tails_Conformational_Dynamics_Kinetics.

## Supporting Information Available

Summary table for system setup (Table S1) and MSM validation CK tests, PCCA+ clustering, and transition probability network plots for all histone tails (Figures S1–S25) (PDF)
