# Supplementary material for: Unveiling the Conformational Dynamics of the Histone Tails Using Markov State Modeling
Source: J Chem Theory Comput. 2025 Apr 28;21(9):4921–38. doi: 10.1021/acs.jctc.5c00196 (PMC12080106; doi:10.1021/acs.jctc.5c00196)
Supplement: Supplementary file 1 — ct5c00196_si_001.pdf [file ct5c00196_si_001.pdf]

Supporting Information

Unveiling the Conformational Dynamics of the Histone Tails Using Markov State Modeling

Rutika Patel[1,2], Sharon M. Loverde[1,2,3,4]

[1]Ph.D. Program in Biochemistry, The Graduate Center of the City University of New York, New York, NY, 10016

[2]Department of Chemistry, College of Staten Island, The City University of New York, 2800 Victory Boulevard, Staten Island, New York, 10314, United States.

[3]Ph.D. Program in Chemistry, The Graduate Center of the City University of New York, New York, NY, 10016

[4]Ph.D. Program in Physics, The Graduate Center of the City University of New York, New York, NY, 10016

*Sharon M. Loverde

Email: sharon.loverde@csi.cuny.edu

This PDF file includes:

Figures S1 to S25
Table S1

Supporting Methods

Markov State Models (MSMs)

Markov State Models (MSMs) approximate the kinetics of molecules as memoryless processes, where the future state of the system depends on its current state and not the previous one. The feature selection to construct the MSMs can be selected based on the VAMP-2 score. Here, we selected a combination of backbone torsions and pairwise distance as the feature. The feature space is further reduced using time-lagged independent component analysis (TICA).

To apply TICA, instantaneous (C (0)) and time-lagged (C((τ)) covariance matrices are computed with elements $C_{ij}(0) = \langle X_i(t)\, X_j\,(t)\rangle_t$ and $C_{ij}(\tau) = \langle X_i(t)\, X_j\,(t+\tau)\rangle_t$ where $X_i(t)$ represents the ith feature at time t after the mean has been removed[1, 2]. A *k*-means clustering algorithm is used to conformationally cluster the TICA projections to define microstates.

The implied timescales (ITS) are determined from the eigenvalues of the transition probability matrix P(τ). Implied timescales MSM validation is done using the Chapman-Kolmogorov (CK) test. are computed as $ti\,(\tau) = -\frac{\tau}{\ln \lambda i}$, where λi is an eigenvalue computed from the transition probability matrix P(τ), and τ is the lag time. The optimal lag time is determined by plotting the ITS as a function of the lag time. The chosen lag time is the smallest possible lag time at which the implied timescales plateau and remain approximately constant, indicating the Markovian nature of the system[2-4].

Further, the MSM validation can be done using the Chapman-Kolmogorov (CK) test. The CK property of the Markovian matrix is $P(k\tau) = P^{\,k}(\tau)$ where the left-hand side of the equation corresponds to an MSM estimated at lag time $(k\tau)$ and *k* is an integer larger than 1. The right-hand side of the equation is the predicted MSM transition probability matrix to the kth power. The MSM is validated based on how well both sides adhere.

After computing the transition probabilities, we also calculate the mean first passage time (MFPT), which provides the mean transition time between the states of the histone tails in the MSM. This equation can determine the MFPT from the initial state i to the final state j. $MFPT_{if} = \sum_j T(\tau)_{ij}\,(\tau + MFPT_{jf})$. Here, τ is the lag time, and T(τ) is the corresponding transition probability matrix. To calculate the MFPT from the microstate MSM, first the MFPTs of all the microstates within the destination macrostate is set to be zero. Then, MFPTs starting from each microstate in the starting macrostate are calculated. A weighted average is obtained as $MFPT_{if}$. $MFPT_{if} = \sum_{l\in i} p_l MFPT_{lf}$, here p_l is the normalized population of the microstate *l* within the macrostate *i*. The kinetic information of the histone tails is extracted from the MSM by calculating the MFPTs between the different macrostates [5, 6]. The inverse of the MFPT (1/mfpt) provides the rate between the transition states.

Supporting Figures

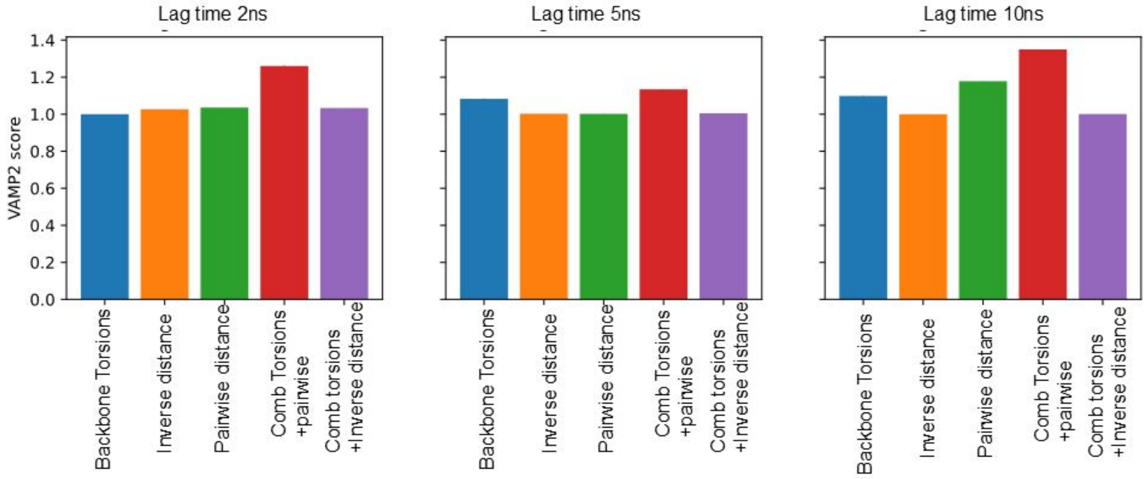

Fig. S1. Feature selection. VAMP-2 scores of the various features of histone tails calculated at various lag times. Backbone (blue), inverse distance (orange), C_α pairwise distances (green), combination of the backbone torsions and pairwise distances (red), and combination of backbone torsions and inverse distance (purple). The combination of backbone torsions and pairwise distances is a superior feature across all various lag times and is used for all analyses.

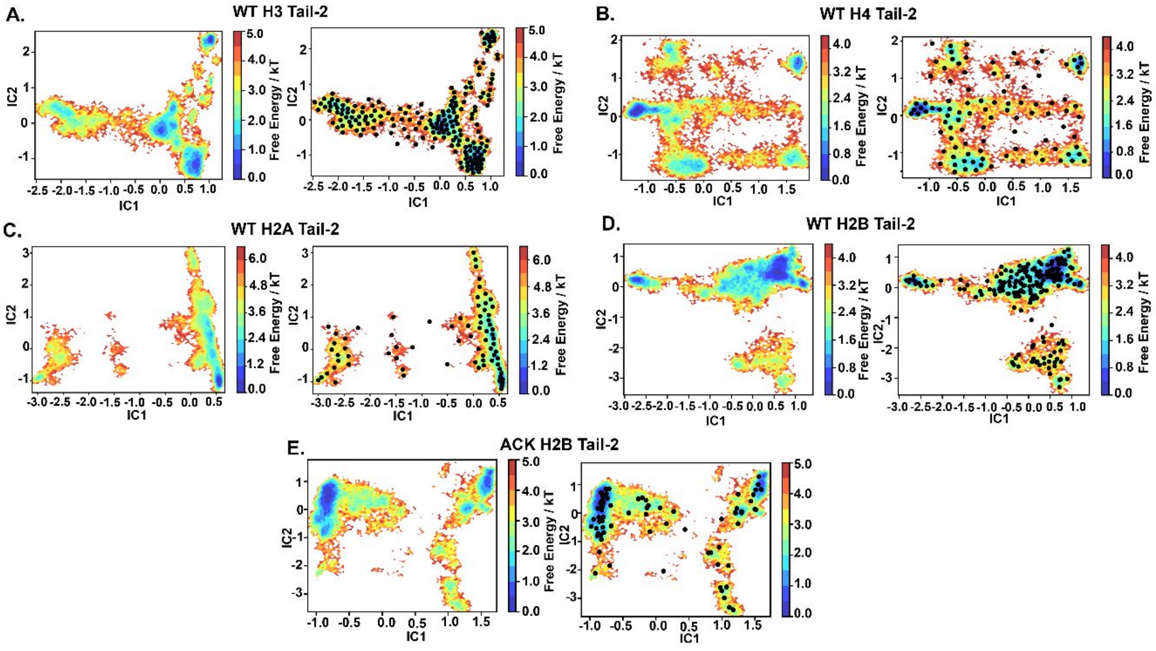

Fig. S2. Dimensionality Reduction using Time Independent Component Analysis (TICA). The free energy surface and *k*-means clustering visualizations are projected onto the leading two Independent Components (ICs) for WT **(A)** H3 tail-2 **(B)** H4 tail-2 **(C)** H2A tail-2 **(D)** H2B tail-2 and **(E)** ACK H2B tail-2. The *k*-means clustering provides microstates for each tail based on their conformational space.

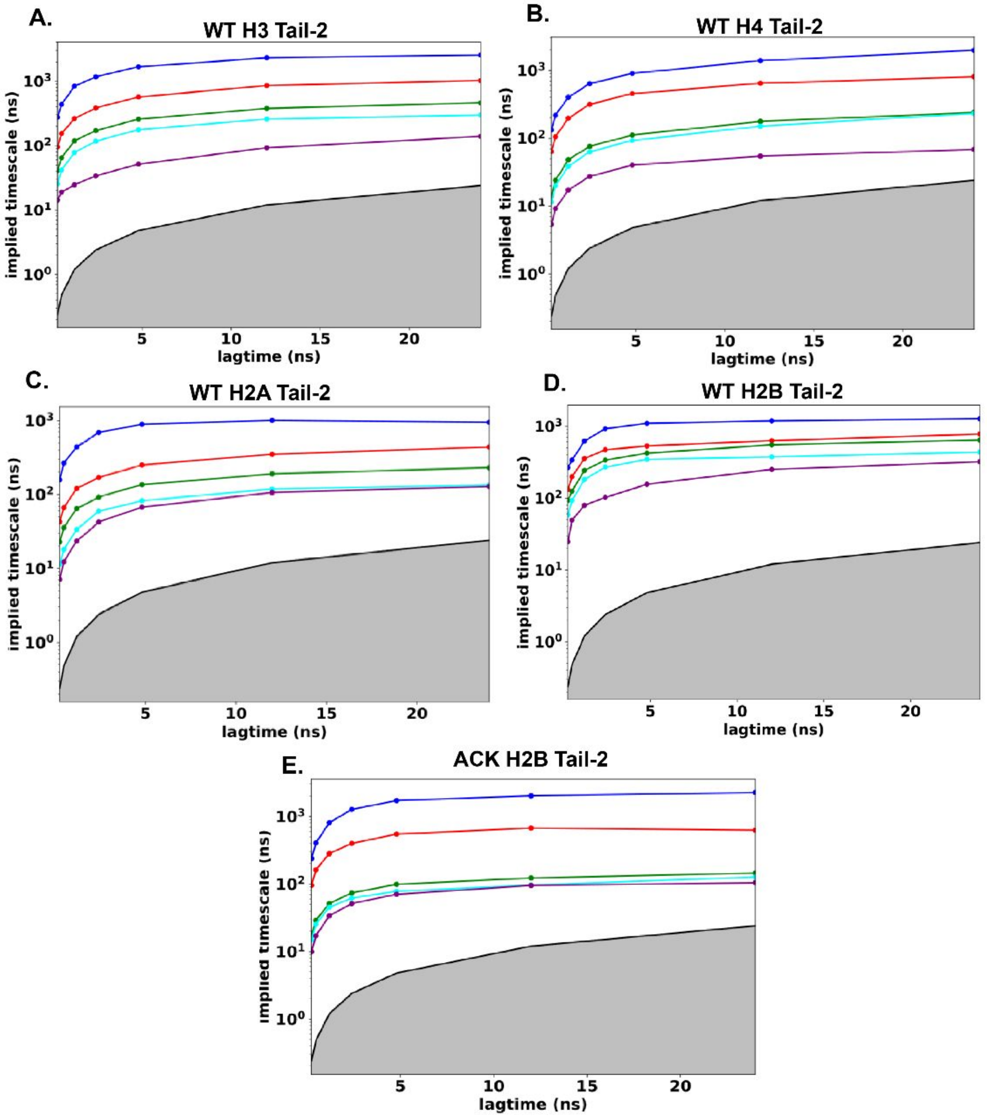

Fig. S3. Implied Timescales of Histone N-terminal tails. The implied timescales (ITs) are associated with the five slowest processes for WT **(A)** H3 tail-2 **(B)** H4 tail-2 **(C)** H2A tail-2 **(D)** H2B tail-2 and **(E)** ACK H2B tail-2. The implied timescales plots show Markov processes with different lag times. The solid line corresponds to the maximum likelihood. The blackline with the grey shaded area indicates the timescale horizon below which the MSM cannot resolve processes.

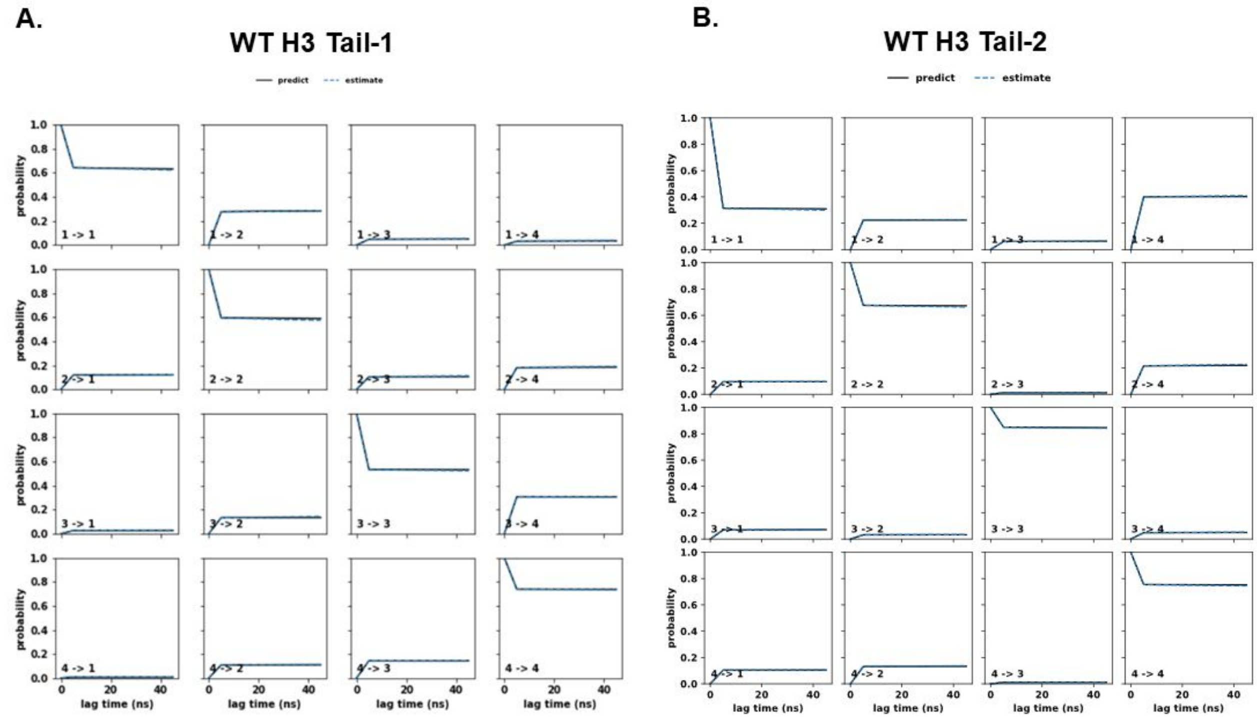

Fig. S4. Chapman-Kolmogorov (CK) test for MSM validation of H3 Tails. (A) H3 tail-1 and **(B)** H3 tail-2 show four metastable states. The predictions from our MSM (blue-dash line) agree well with the MSM estimated (solid line) for all states.

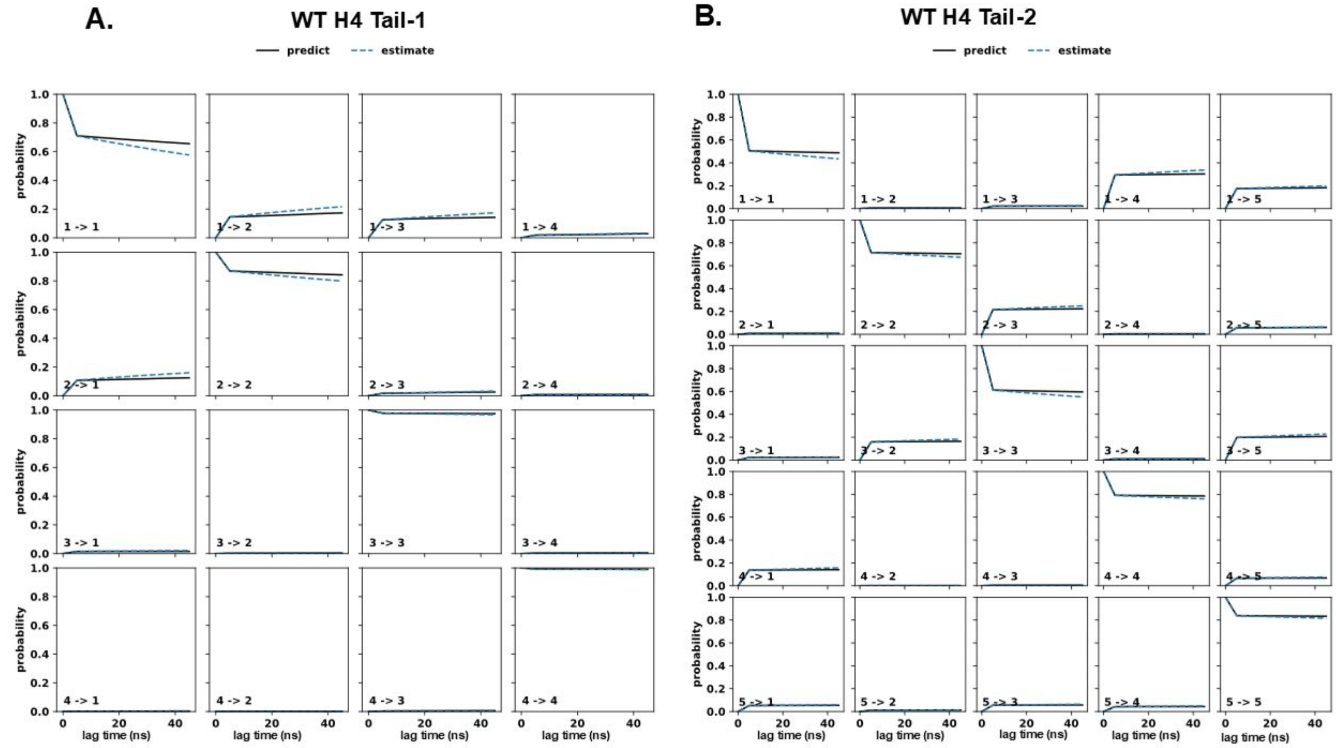

Fig. S5. Chapman-Kolmogorov (CK) test for MSM validation of H4 Tails. (A) H4 tail-1 and **(B)** H4 tail-2 show four and five metastable states, respectively. The predictions from our MSM (blue-dash line) agree well with the MSM estimated (solid line) for all states.

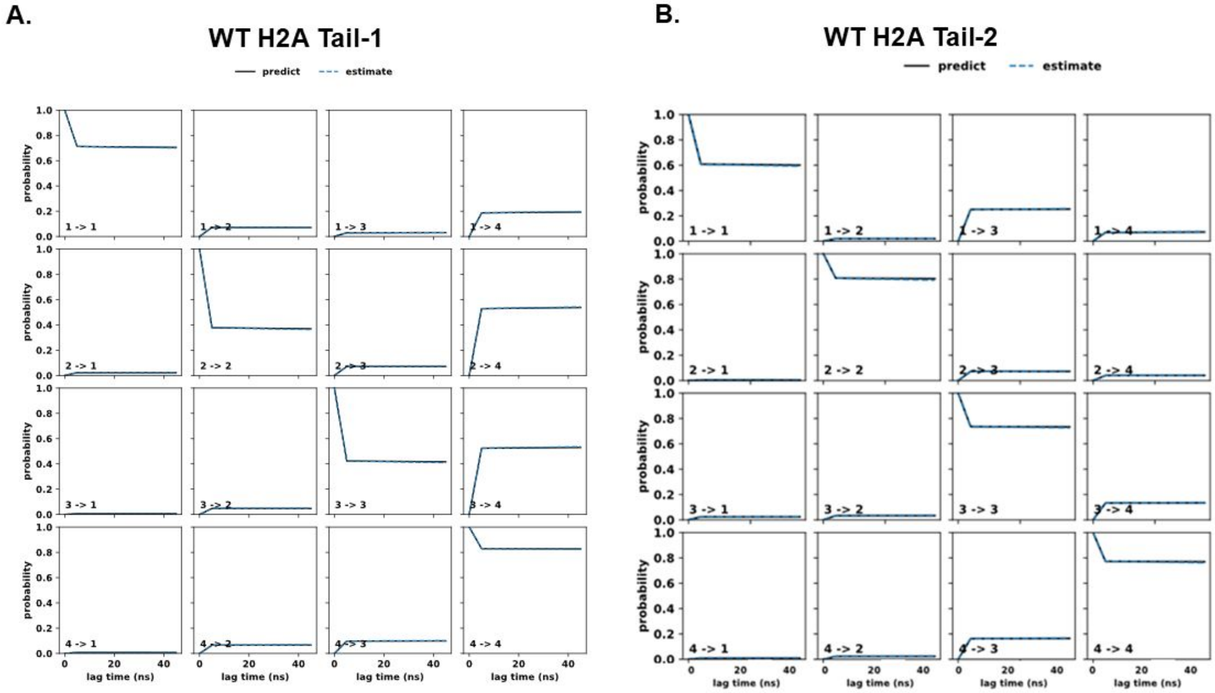

Fig. S6. Chapman-Kolmogorov (CK) test for MSM validation of H2A Tails. (A) H2A tail-1 and **(B)** H2A tail-2 show four metastable states. The predictions from our MSM (blue-dash line) agree well with the MSM estimated (solid line) for all states.

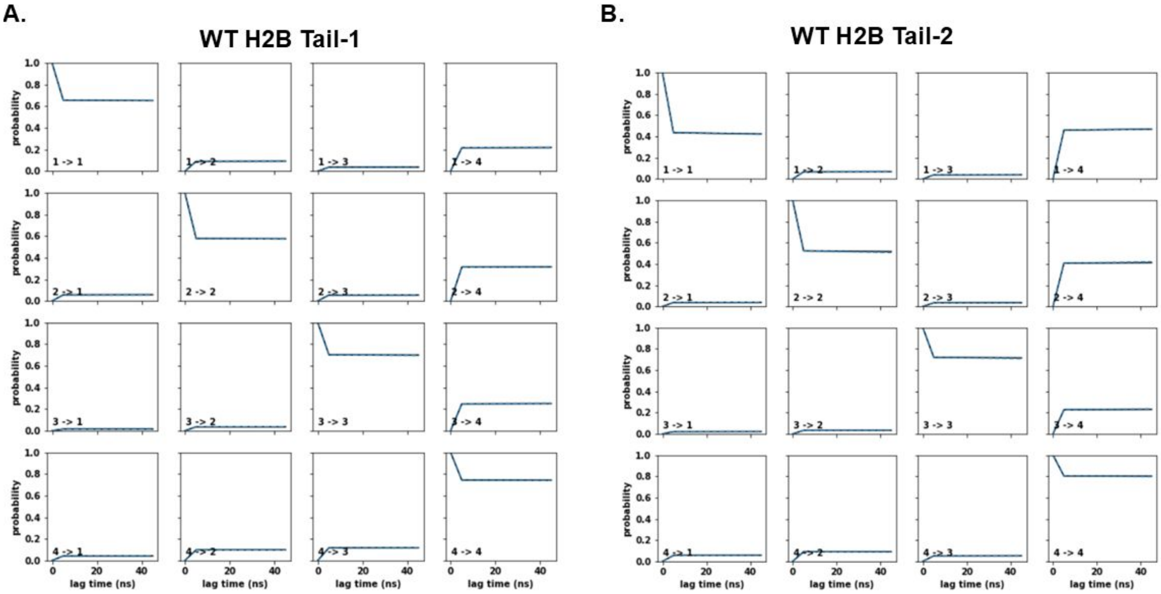

Fig. S7. Chapman-Kolmogorov (CK) test for MSM validation of H2B Tails. (A) WT H2B tail-1 and **(B)** WT H2B tail-2 show four and five metastable states, respectively. The predictions from our MSM (blue-dash line) agree well with the MSM estimated (solid line) for all states.

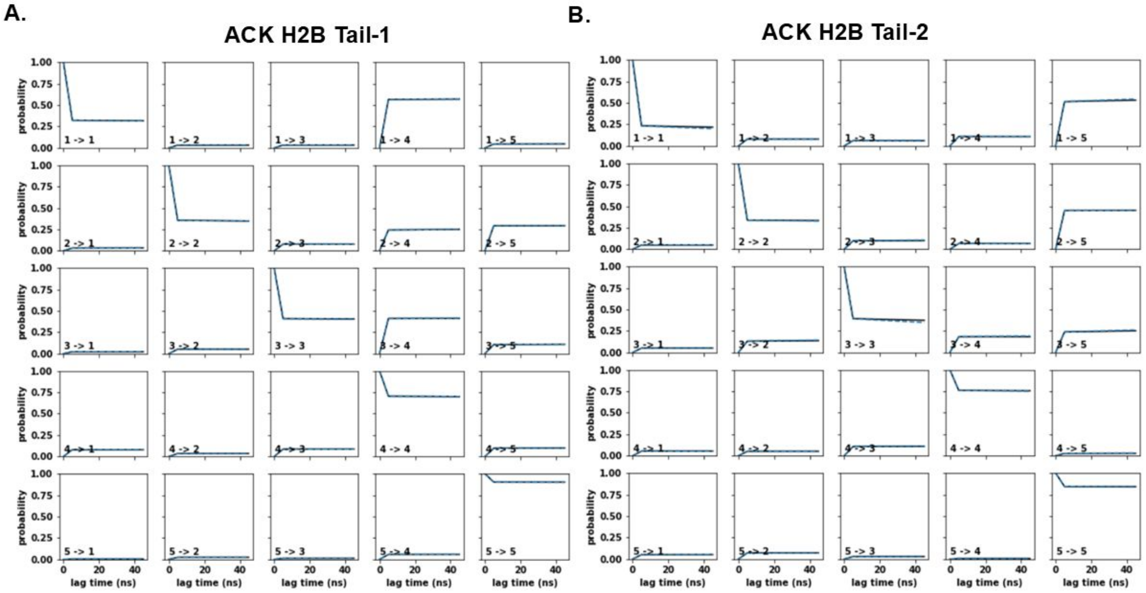

Fig. S8. Chapman-Kolmogorov (CK) test for MSM validation of ACK H2B Tails. (A) ACK H2B tail-1 and **(B)** ACK H2B tail-2 show four and five metastable states, respectively. The predictions from our MSM (blue-dash line) agree well with the MSM estimated (solid line) for all states.

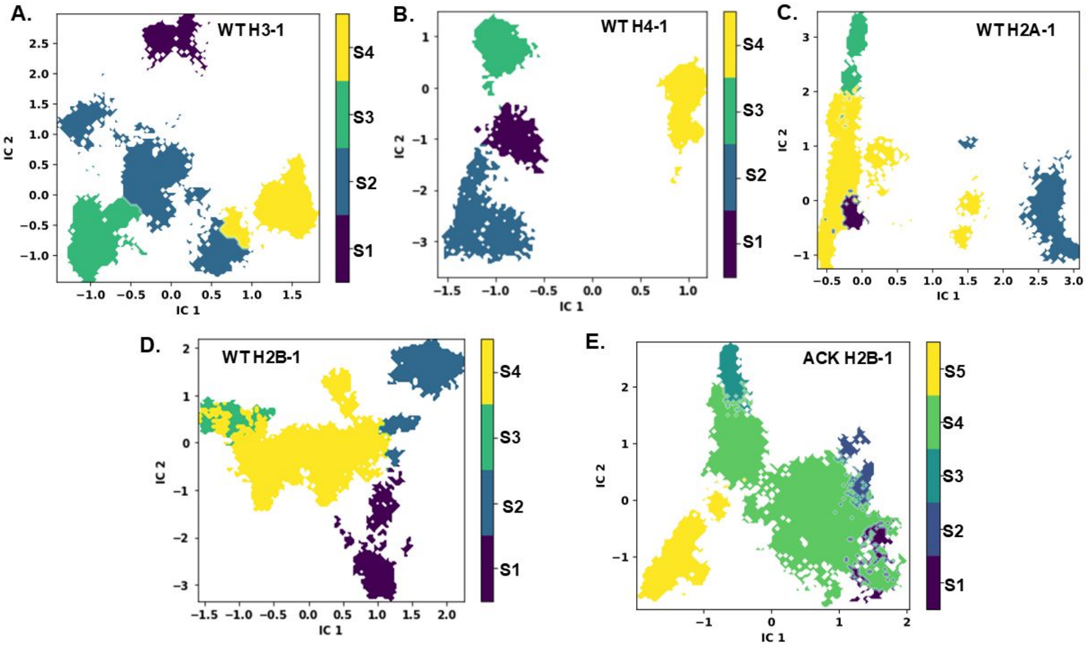

Fig. S9. Macrostate PCCA+ Clustering Histone Tails-1. The macrostate clustering visualization projected onto the leading TICA coordinates for visualization of different macrostates for WT **(A)** H3 **(B)** H4 **(C)** H2A **(D)** H2B and **(E)** ACK H2B tails. The metastable macrostates are computed by the PCCA+ clustering algorithm within the same TICA projections.

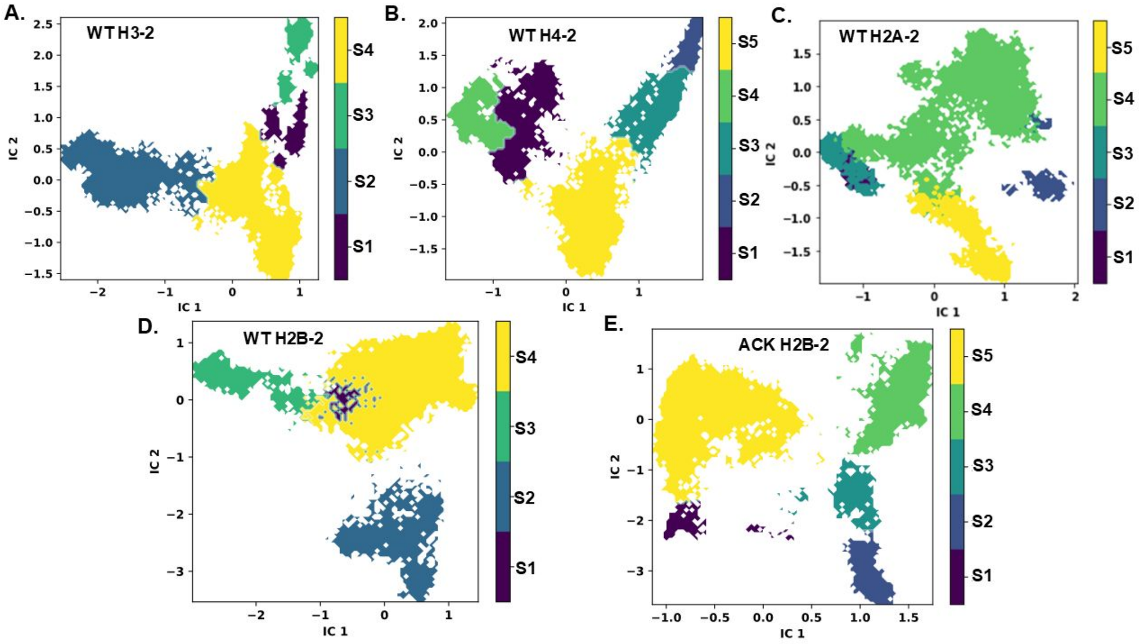

Fig. S10. Macrostate PCCA+ Clustering Histone Tails-2. The macrostate clustering visualization projected onto the leading TICA coordinates for visualization of different macrostates for WT **(A)** H3 **(B)** H4 **(C)** H2A **(D)** H2B and **(E)** ACK H2B tails. The metastable macrostates are computed by the PCCA+ clustering algorithm within the same TICA projections.

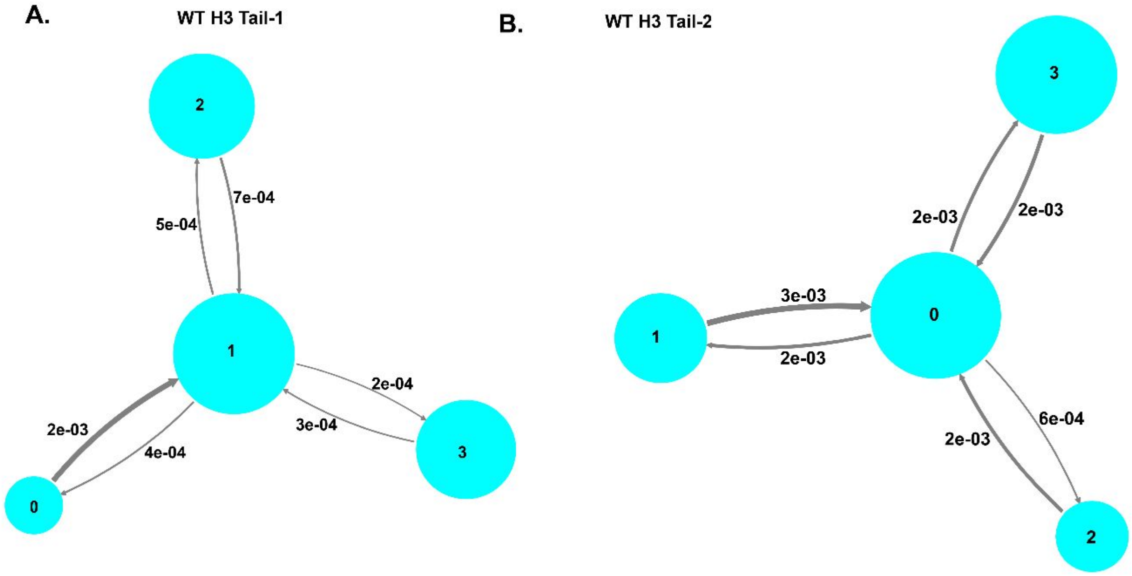

Fig. S11. MSM Transition probability network plot of H3 Tails. (A) and **(B)** The MSM network plot connects four macrostates for WT H3 tail-1 and tail-2 represented as cyan circles. The sizes of the circles are proportional to their stationary population for each state. The states are connected through arrows with their transition probability from one state to another is shown next to arrows. The thickness of the arrows is proportional to the amount of flux between the transition of macrostates.

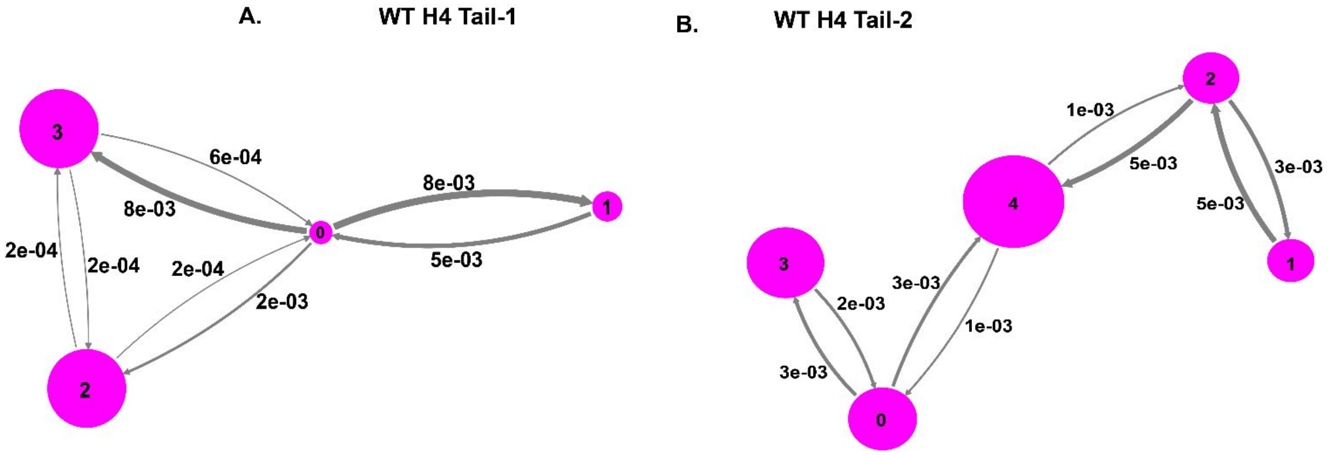

Fig. S12. MSM Transition probability network plot of H4 Tails. (A) and **(B)** The MSM network plot connects four and five macrostates for WT H4 tail-1 and tail-2 represented as magenta circles. The sizes of the circles are proportional to their stationary population for each state. The states are connected through arrows with their transition probability from one state to another is shown next to arrows. The thickness of the arrows is proportional to the amount of flux between the transition of macrostates.

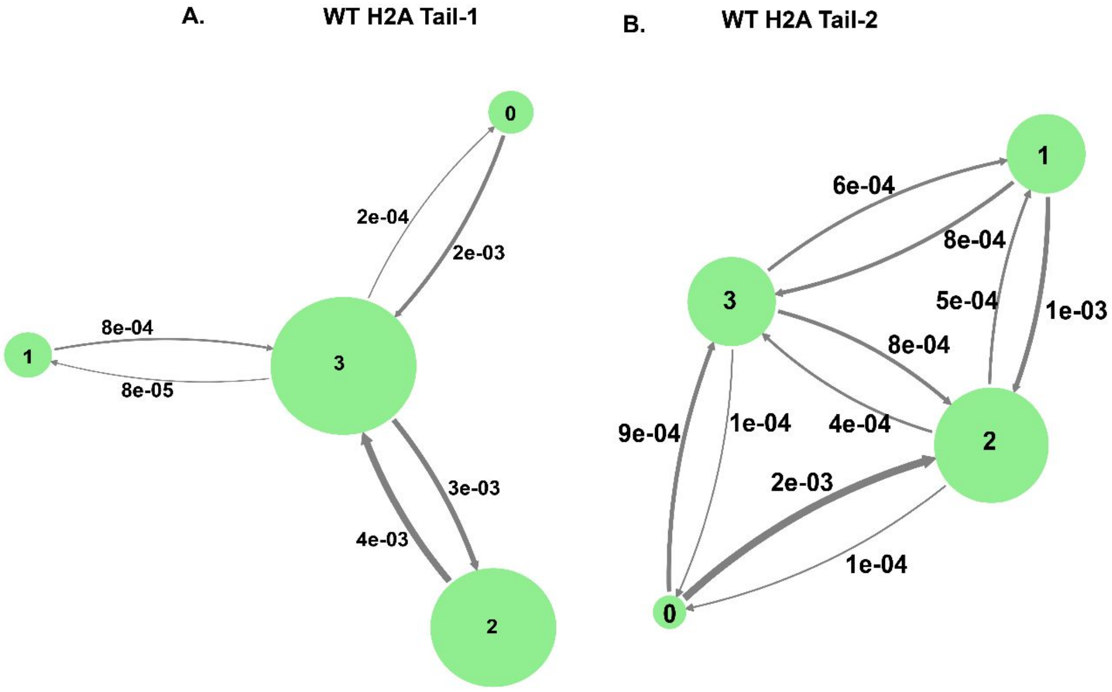

Fig. S13. MSM Transition probability network plot of H2A Tails. (A) and **(B)** The MSM network plot connects four macrostates for WT H2A tail-1 and tail-2 represented as green circles. The sizes of the circles are proportional to their stationary population for each state. The states are connected through arrows with their transition probability from one state to another is shown next to arrows. The thickness of the arrows is proportional to the amount of flux between the transition of macrostates.

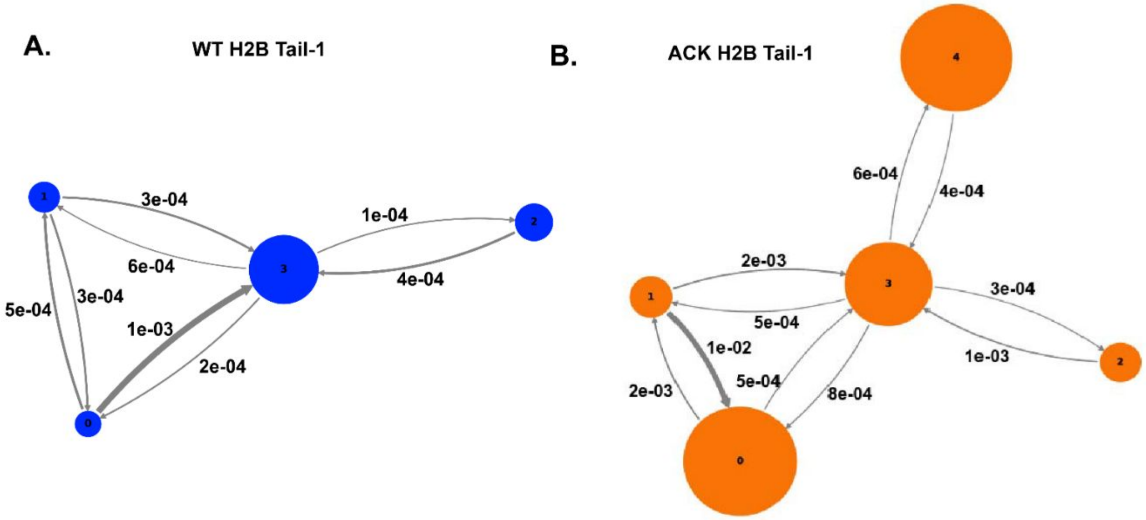

Fig. S14. MSM Transition probability network plot of H2B Tails. (A) and **(B)** The MSM network plot connects four macrostates for WT and five for ACK system represented as blue and orange circles respectively. The sizes of the circles are proportional to their stationary population for each state. The states are connected through arrows with their transition probability from one state to another is shown next to arrows. The thickness of the arrows is proportional to the amount of flux between the transition of macrostates.

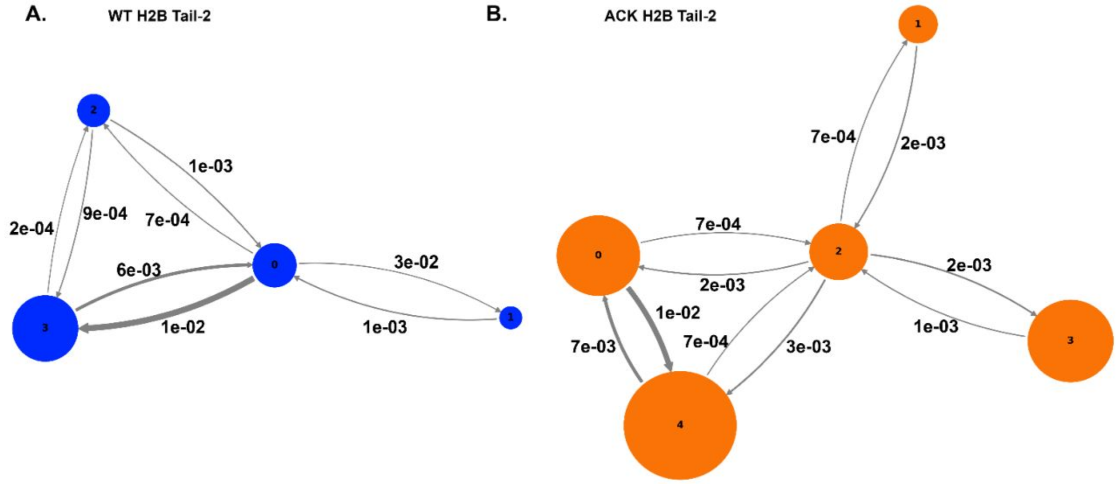

Fig. S15. MSM Transition probability network plot of H2B Tails. (A) and **(B)** The MSM network plot connects four macrostates for WT and five for ACK system represented as blue and orange circles respectively. The sizes of the circles are proportional to their stationary population for each states. The states are connected through arrows with their transition probability from one state to another is shown next to arrows. The thickness of the arrows is proportional to the amount of flux between the transition of macrostates.

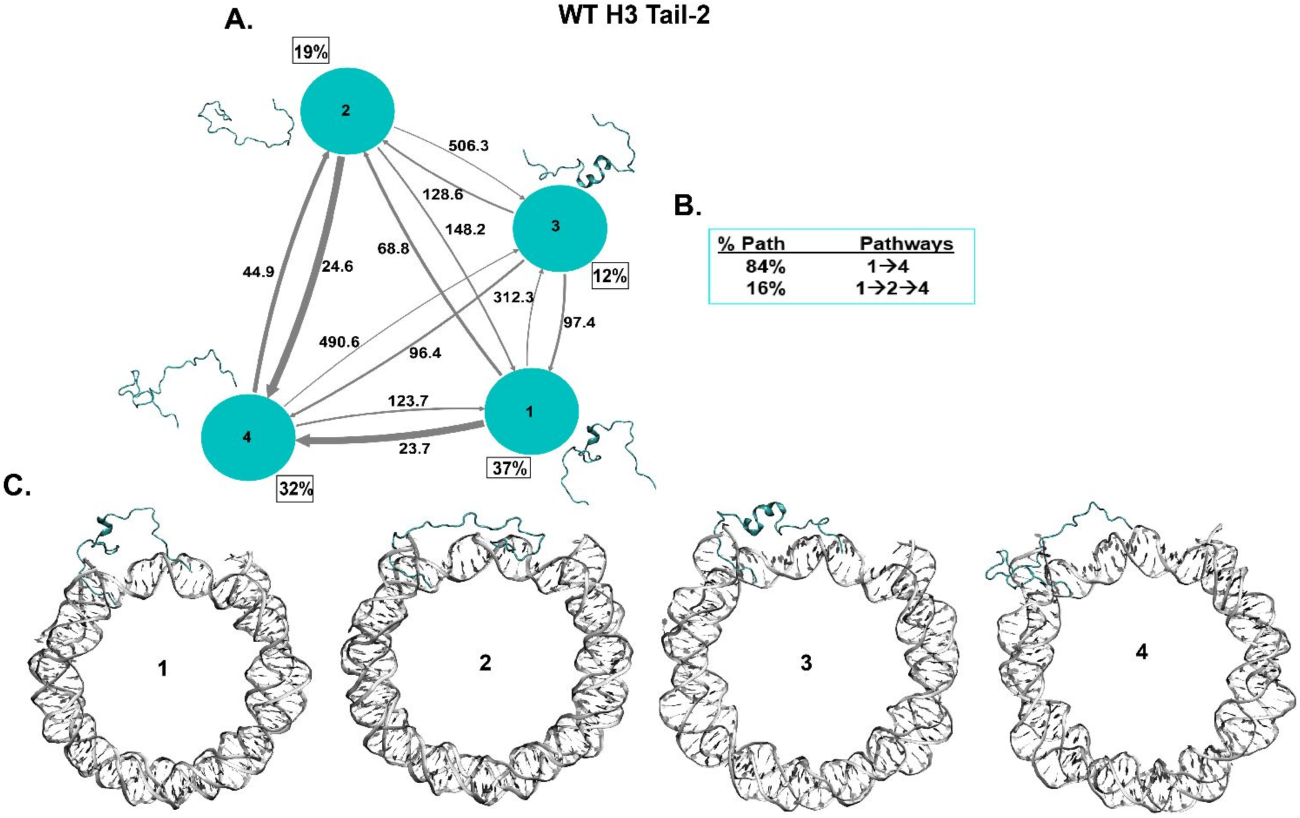

Fig. S16. The Kinetic network of conformational states of H3 Tail-2. (A) WT H3 Tail-2 (cyan circles) network plots connect four macrostates of H3 tail-2. The corresponding conformations of each states are shown next to each state for H3 tail-2. The population percentages of each conformation are shown next to each state. The macrostates are connected with arrows. The thickness of the arrows is proportional to the rate of the transition and is labelled with its respective MFPT values in nanoseconds. **(B)** The net flux of the network is obtained from the Transition Path Theory (TPT) analysis for H3 tail-2. These TPT calculations show major pathways with their path percentages for H3 tail-2. **(C)** The conformational states of H3 tail-2 with NCP DNA are shown. It shows the position of the tail (cyan) at each macrostate with respect to DNA (silver).

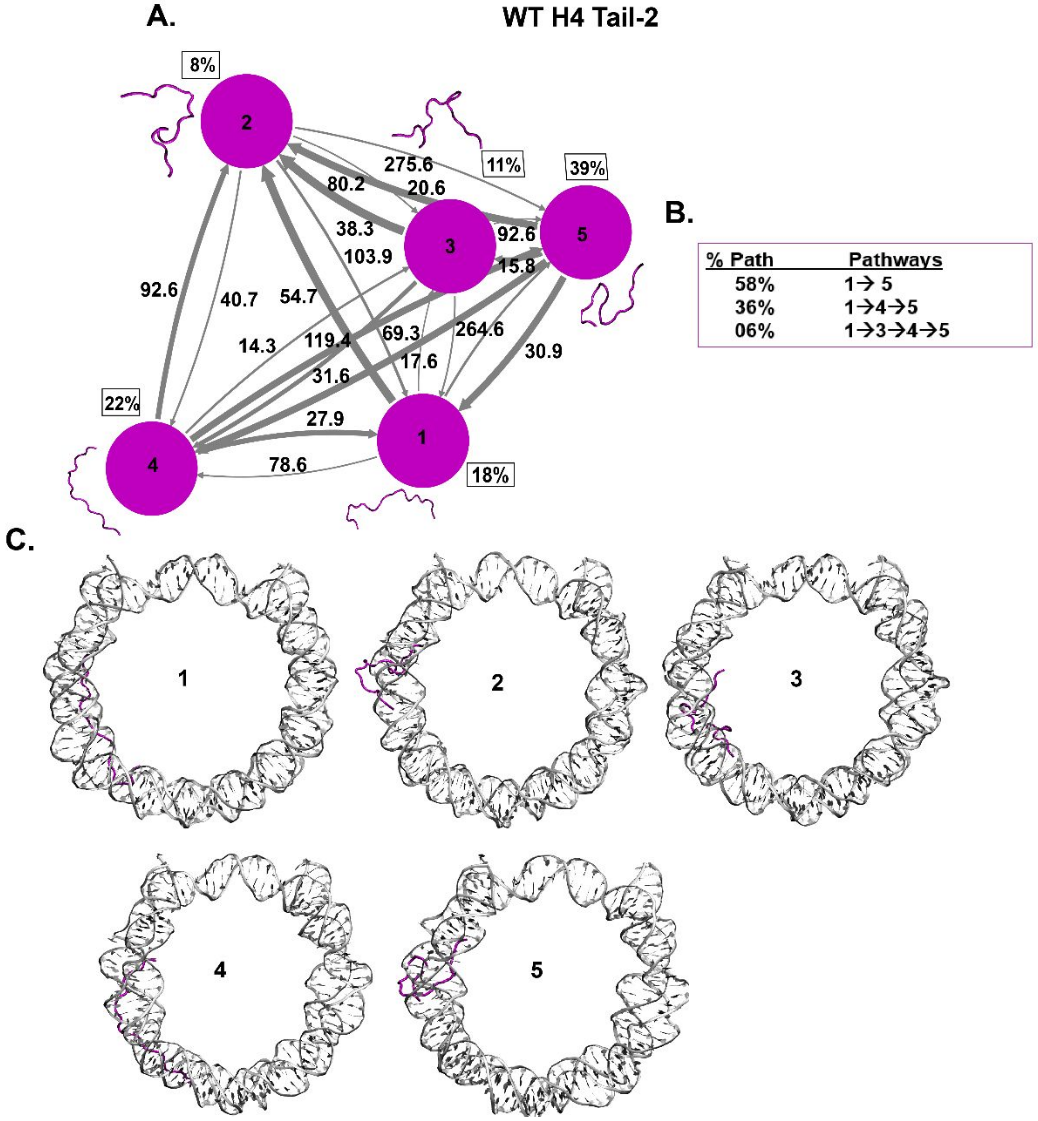

Fig. S17. The Kinetic network of conformational states of H4 Tail-2. (A) WT H4 Tail-2 (magenta circles) network plots connect five macrostates of H4 tail-2. The corresponding conformations of each state are shown next to each state for H4 tail-2. The population percentages of each conformation are shown next to each state. The macrostates are connected with arrows. The thickness of the arrows is proportional to the rate of the transition and is labelled with its respective MFPT values in nanoseconds. **(B)** The net flux of the network is obtained from Transition Path Theory (TPT) analysis for H4 tail-2. These TPT calculations show major pathways with their path percentages for H4 tail-2. **(C)** The conformational states of H4 tail-2 with NCP DNA are shown. It shows the position of the tail (magenta) at each macrostates with respect to DNA (silver).

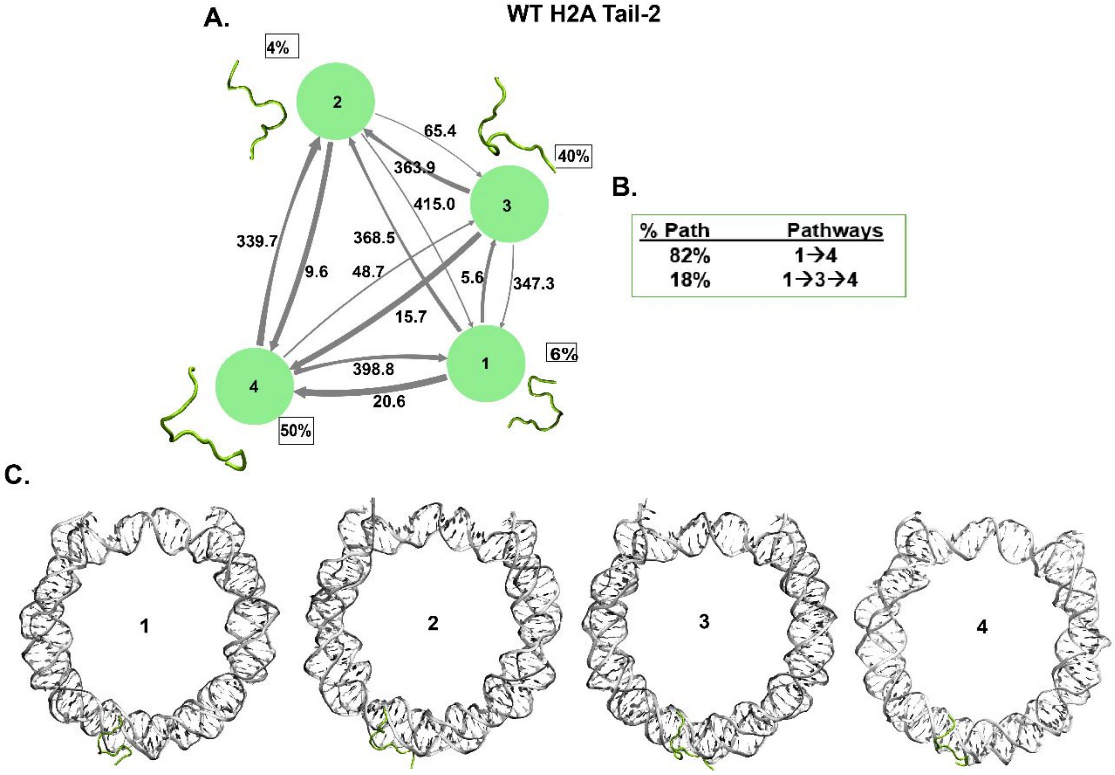

Fig. S18. The Kinetic network of conformational states of H2A Tail-2. (A) WT H2A Tail-2 (green circles) network plots connect four macrostates of H2A tail-2. The corresponding conformations of each state are shown next to each state for H2A tail-2. The population percentages of each conformation are shown next to each state. The macrostates are connected with arrows. The thickness of the arrows is proportional to the rate of the transition and is labelled with its respective MFPT values in nanoseconds. **(B)** The net flux of the network is obtained from Transition Path Theory (TPT) analysis for H2A tail-2. These TPT calculations show major pathways with their path percentages for H2A tail-2. **(C)** The conformational states of H2A tail-2 with NCP DNA are shown. It shows the position of the tail (green) at each macrostates with respect to DNA (silver).

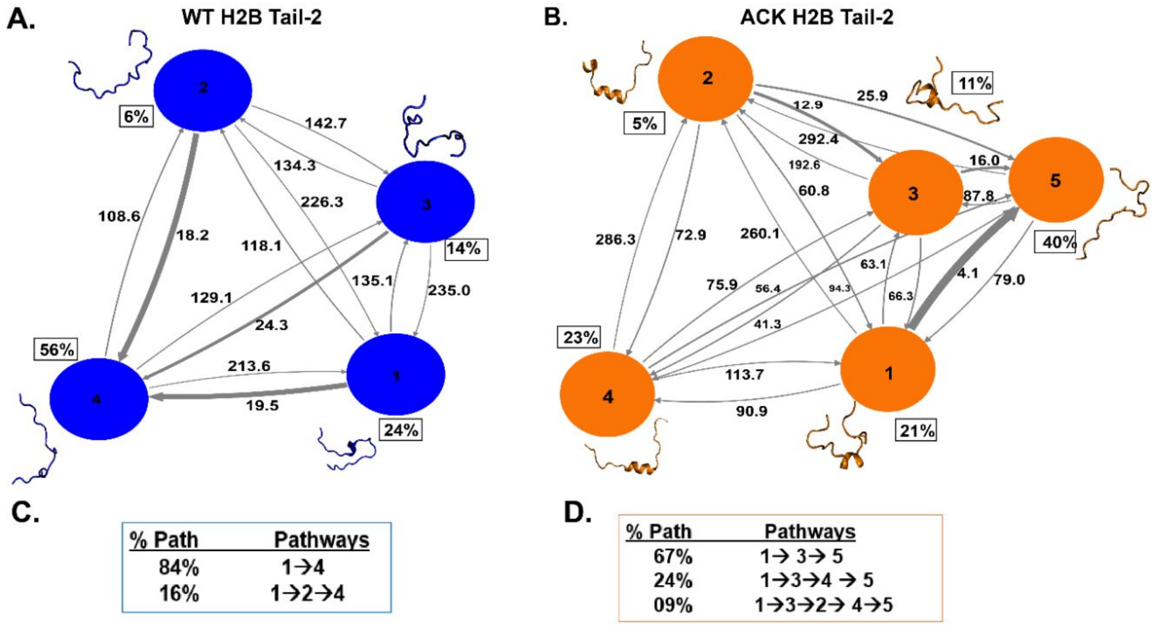

Fig. S19. The Kinetic network of conformational states of H2B Tail-2. (A) and **(B)** WT (blue circles) and ACK (orange circles) H2B Tail-1 network plots connect four and five macrostates, respectively, for H2B tail-2. The corresponding conformations of each state are shown next to each state for both WT (blue) and ACK (orange) H2B tail-2. The population percentages of each conformation are shown next to each state. The macrostates are connected with arrows. The thickness of the arrows is proportional to the rate of the transition and is labelled with its respective MFPT values in nanoseconds. **(C)** and **(D)** The net flux of the network is obtained from Transition Path Theory (TPT) analysis for both WT and ACK H2B tail-2. These TPT calculations show major pathways with their path percentages for both WT and ACK H2B tail-2 systems.

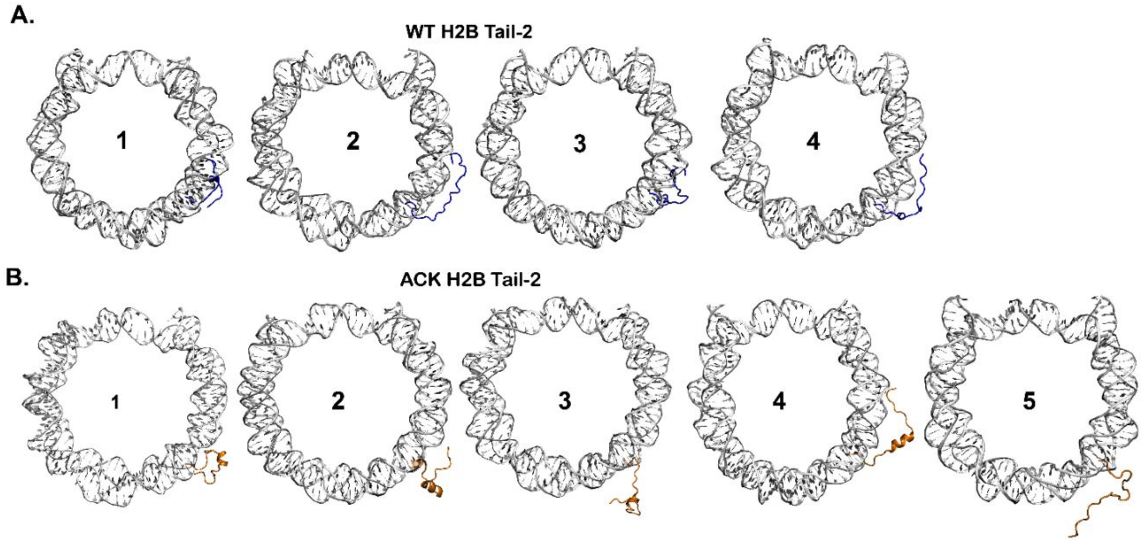

Fig. S20. The conformational states of H2B Tail-2 with DNA of NCP. (A) and **(B)** WT (blue) and ACK (orange) H2B Tail-2 conformational states that are shown in Figure 6, the same conformational states of the tail with NCP DNA are shown here. It shows the position of each tail conformation at each macrostates shown earlier with regard to the DNA, whether the tail collapsed to the DNA or elongated outwards from the DNA.

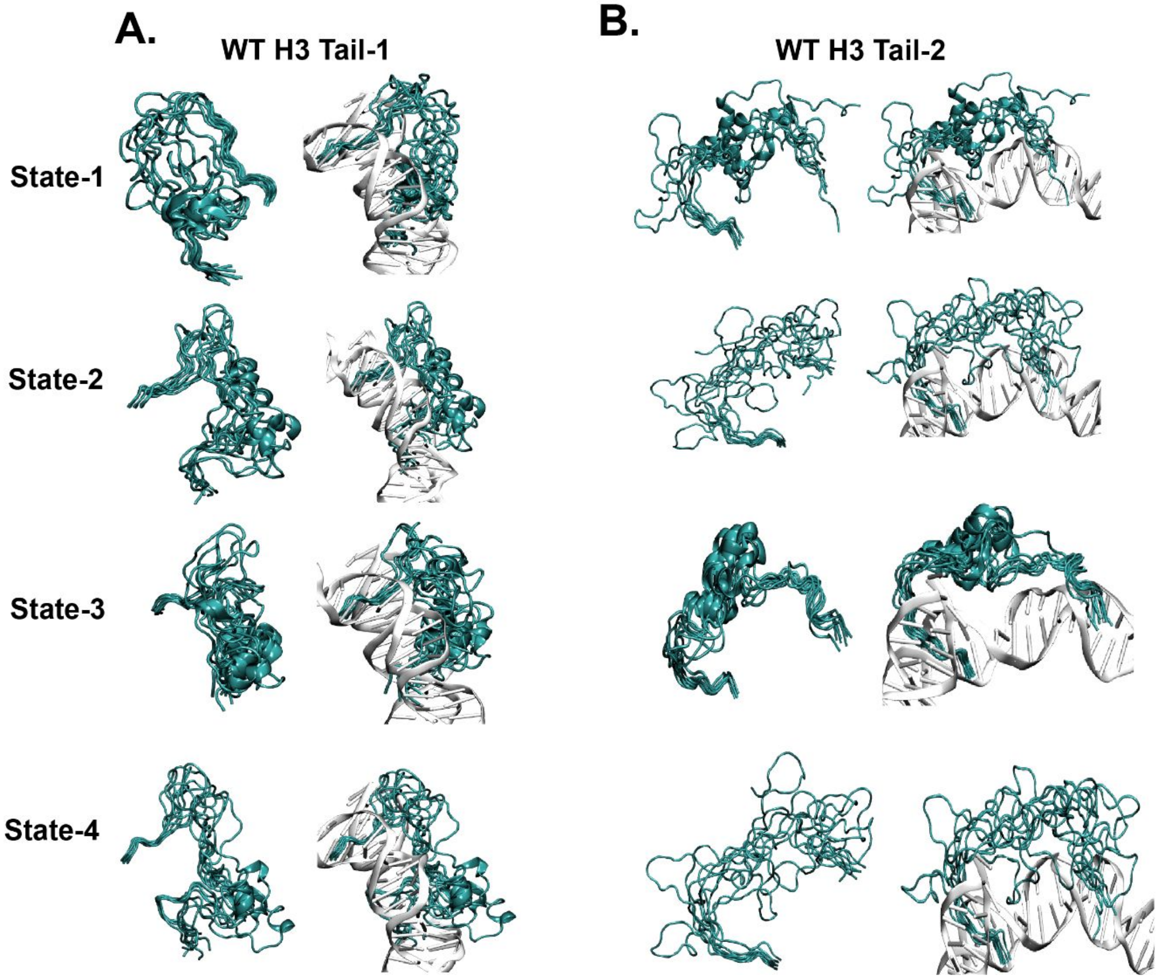

Fig. S21. Histone H3. The conformations of each state for H3 tail (A) tail-1 and (B) tail 2. Each state represents an overlay of multiple tail structures (cyan) with one surface representation of nucleosomal DNA (silver).

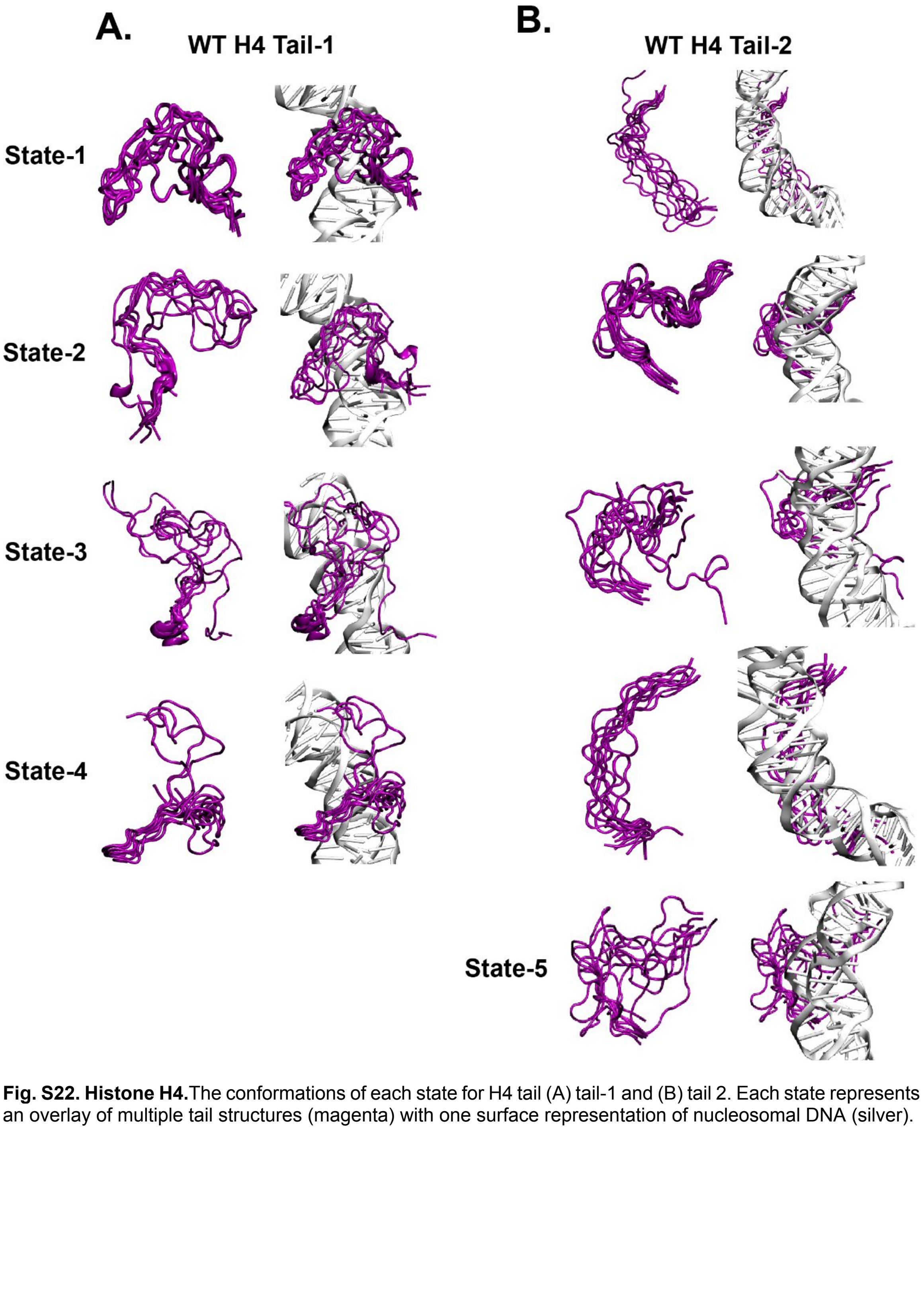

Fig. S22. Histone H4. The conformations of each state for H4 tail (A) tail-1 and (B) tail 2. Each state represents an overlay of multiple tail structures (magenta) with one surface representation of nucleosomal DNA (silver).

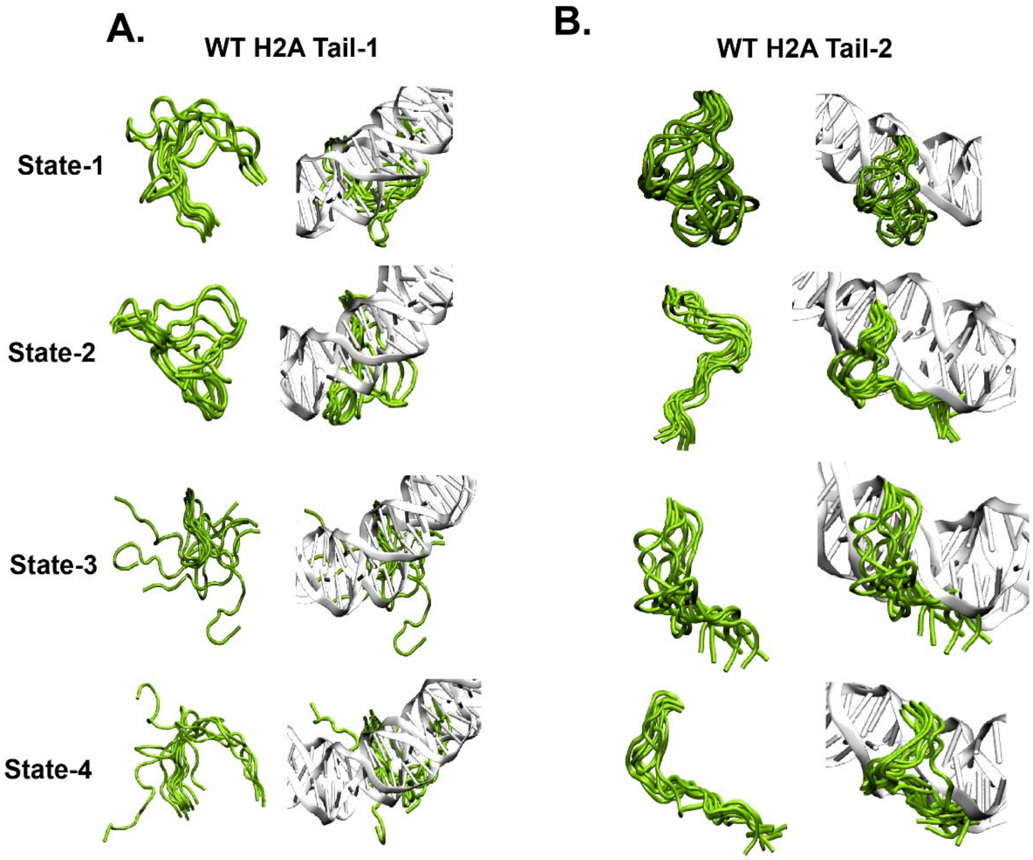

Fig. S23. Histone H2A. The conformations of each state for H2A tail (A) tail-1 and (B) tail 2. Each state represents an overlay of multiple tail structures (green) with one surface representation of nucleosomal DNA (silver).

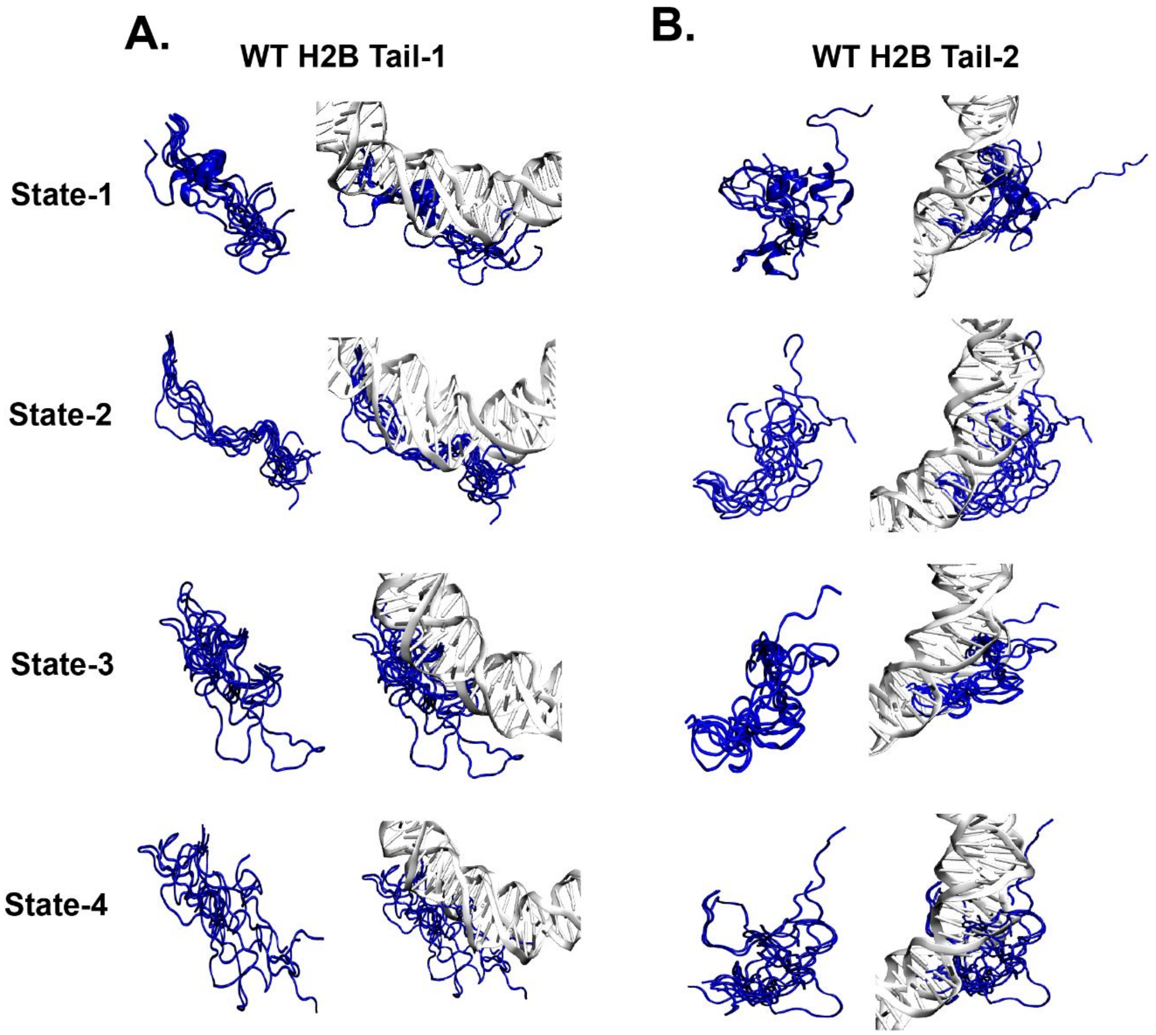

Fig. S24. Histone H2B. The conformations of each state for H2B tail (A) tail-1 and (B) tail 2. Each state represents an overlay of multiple tail structures (blue) with one surface representation of nucleosomal DNA (silver).

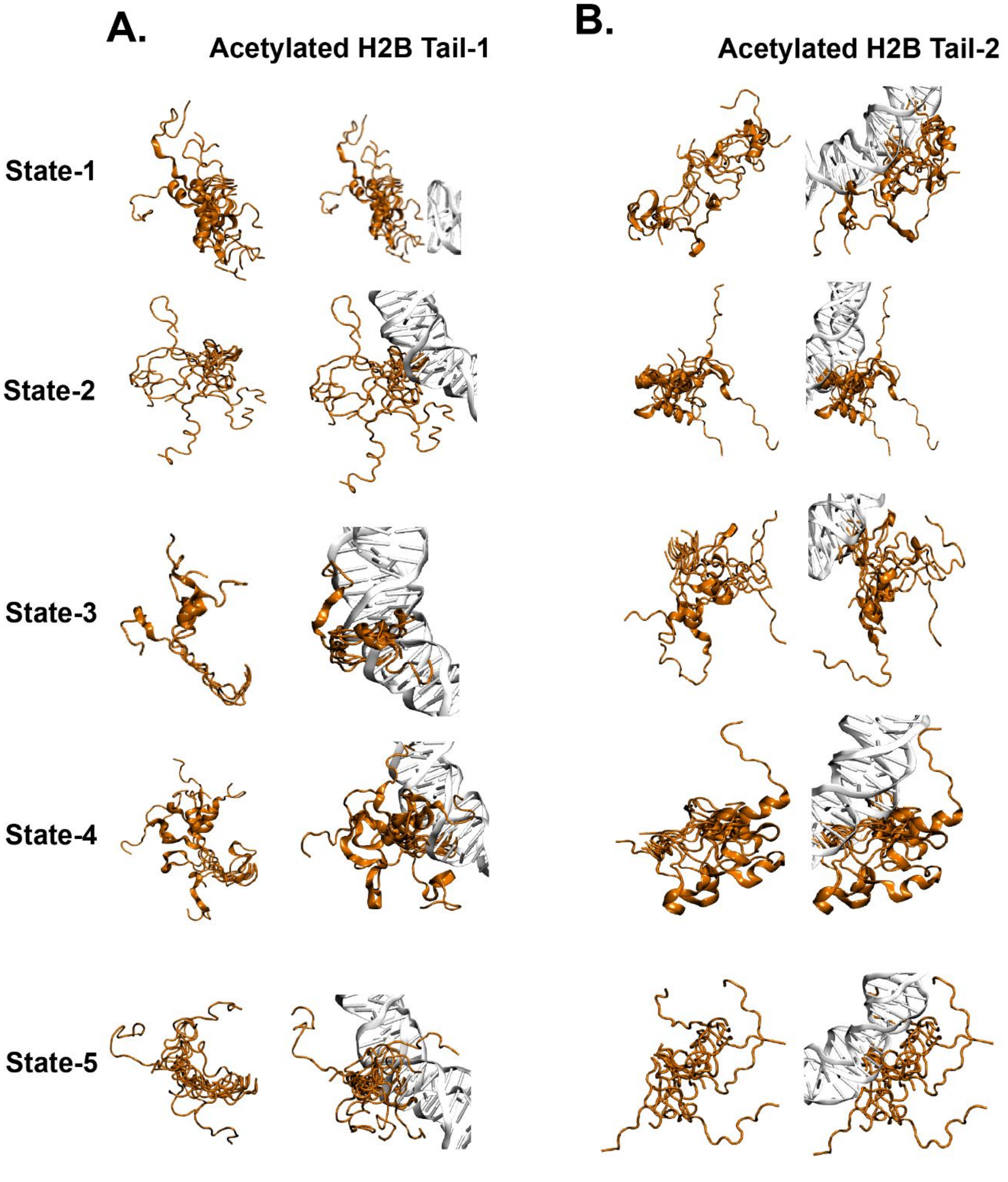

Fig. S25. Histone H2B Acetylated. The conformations of each state for acetylated H2B tail (A) tail-1 and (B) tail 2. Each state represents an overlay of multiple tail structures (orange) with one surface representation of nucleosomal DNA (silver).

Tables.

Table S1. Summary of NCP systems MD simulation set up box sizes and atoms/ions.

NCP systems	WT_0.15M (unacetylated)	ACK_0.15M (acetylated)
**Box Size (Å^3)**	159 x 191 x 112	159 x 191 x 112
**No. of atoms**	444888	486492
**No. of Water molecules**	104740	115117
**No. of Na^+ ions**	472	508
**No. of Cl^- ions**	356	384
**No. of Mg^{2+} ions**	14	14
**NaCl salt Concentration (M)**	0.15	0.15

References

(1) Noé, F.; Clementi, C. Kinetic Distance and Kinetic Maps from Molecular Dynamics Simulation. *Journal of Chemical Theory and Computation* **2015**, *11*, 5002-5011. DOI: 10.1021/acs.jctc.5b00553.

(2) Wehmeyer, C.; Scherer, M. K.; Hempel, T.; Husic, B. E.; Olsson, S.; Noé, F. Introduction to Markov State Modeling with the Pyemma Software [Article V1.0]. *Living Journal of Computational Molecular Science* **2019**, *1*, 5965. DOI: 10.33011/livecoms.1.1.5965 (acccessed 2024/10/18).

(3) Swope, W. C.; Pitera, J. W.; Suits, F. Describing Protein Folding Kinetics by Molecular Dynamics Simulations. 1. Theory. *The Journal of Physical Chemistry B* **2004**, *108*, 6571-6581. DOI: 10.1021/jp037421y.

(4) Bowman, G. R.; Pande, V. S.; Noé, F. *An Introduction to Markov State Models and Their Application to Long Timescale Molecular Simulation*; Springer Science & Business Media, 2013.

(5) Zheng, Y.; Cui, Q. The Histone H3 N-Terminal Tail: A Computational Analysis of the Free Energy Landscape and Kinetics. *Phys Chem Chem Phys* **2015**, *17*, 13689-13698. DOI: 10.1039/c5cp01858g From NLM.

(6) Singhal, N.; Snow, C. D.; Pande, V. S. Using Path Sampling to Build Better Markovian State Models: Predicting the Folding Rate and Mechanism of a Tryptophan Zipper Beta Hairpin. *J Chem Phys* **2004**, *121*, 415-425. DOI: 10.1063/1.1738647 From NLM.
